# Anti-fouling graphene-based membranes for effective water desalination

**DOI:** 10.1038/s41467-018-02871-3

**Published:** 2018-02-14

**Authors:** Dong Han Seo, Shafique Pineda, Yun Chul Woo, Ming Xie, Adrian T. Murdock, Elisa Y. M. Ang, Yalong Jiao, Myoung Jun Park, Sung Il Lim, Malcolm Lawn, Fabricio Frizera Borghi, Zhao Jun Han, Stephen Gray, Graeme Millar, Aijun Du, Ho Kyong Shon, Teng Yong Ng, Kostya (Ken) Ostrikov

**Affiliations:** 1 CSIRO Manufacturing, 36 Bradfield Road, Lindfield, NSW 2070 Australia; 2 School of Physics, University of Sydney, Sydney, NSW 2006 Australia; 3 Centre for Technology in Water and Wastewater, School of Civil and Environmental Engineering, University of Technology Sydney, PO Box 123, 15 Broadway, Sydney, NSW 2007 Australia; 4 Institute for Sustainability and Innovation, College of Engineering and Science, Victoria University, Werribee, VIC 3030 Australia; 5 School of Mechanical and Aerospace Engineering, Nanyang Technological University, 50 Nanyang Avenue, Singapore, 639798 Singapore; 6 Institute for Future Environments and Institute for Health and Biomedical Innovation, School of Chemistry, Physics, and Mechanical Engineering, Queensland University of Technology, Brisbane, QLD 4000 Australia; 7 National Measurement Institute, Nanometrology, 36 Bradfield Road, Lindfield, NSW 2070 Australia

## Abstract

The inability of membranes to handle a wide spectrum of pollutants is an important unsolved problem for water treatment. Here we demonstrate water desalination via a membrane distillation process using a graphene membrane where water permeation is enabled by nanochannels of multilayer, mismatched, partially overlapping graphene grains. Graphene films derived from renewable oil exhibit significantly superior retention of water vapour flux and salt rejection rates, and a superior antifouling capability under a mixture of saline water containing contaminants such as oils and surfactants, compared to commercial distillation membranes. Moreover, real-world applicability of our membrane is demonstrated by processing sea water from Sydney Harbour over 72 h with macroscale membrane size of 4 cm^2^, processing ~0.5 L per day. Numerical simulations show that the channels between the mismatched grains serve as an effective water permeation route. Our research will pave the way for large-scale graphene-based antifouling membranes for diverse water treatment applications.

## Abstract (web summary)

Intrinsic limitations of nanoporous graphene limit its applications in water treatment. Here the authors produce post-treatment-free, low-cost graphene-based membranes from renewable biomass and demonstrate their high water permeance and antifouling properties using real seawater.

## Introduction

Graphene, an atomically thin carbon-based two-dimensional material, shows promise for a new class of ultrathin membranes that have atomically defined nanopores with diameters approaching those of hydrated ions. This characteristic is ideal for the design of membranes capable of ultrafast and selective water transport. A pristine single layer of graphene is impermeable to standard gases (e.g., helium)^1^, which motivates the introduction of selective defects throughout the graphene lattice to enable permeance of water molecules. Recent advances in post-synthesis reactive processing of chemical vapour deposition (CVD) graphene have produced atomically thin permeable films for water purification by creating randomly etched pores and nanoscale apertures in the graphene film^2–4^. However, these techniques involve a series of highly controlled, resource-intensive and complex procedures that are difficult to uniformly implement in high density and large scales. Thus, the capability of CVD graphene films for water purification and desalination remains restricted to small-scale demonstrations (µm scale)^2^. Moreover, while CVD synthesis offers good control over the growth of graphene films, it remains an expensive process because of the necessity of compressed gases and extensive vacuum operation. Furthermore, often, hydrophobic nature of CVD graphene creates additional hurdles for utilisation in water purification membrane. Consequently, these technical challenges impede the commercial viability of CVD graphene films for water purification^5^.

MD is an rapidly emerging thermally driven water purification technology that is particularly promising for the treatment of seawater, industrial effluents and brine obtained from reverse osmosis and various desalination processes^6^. In the MD process, water purification is driven by a vapour pressure gradient across a porous and hydrophobic membrane. This situation is created by parallel flows of a hot feed solution and permeate stream, where water vapour is formed at the interface of the membrane’s hot feed side and is transported to the opposing cold permeate side^7^. Key advantageous features of the MD process include water production almost independent of the feed solution salinity, and the potential to reject majority of non-volatile constituents, such as dissolved salt, organics, colloids and the ability to utilise low-grade waste heat to drive the process. These merits enable MD to be a promising green technology for zero liquid discharge purification processes^8^.

MD has few but important major drawbacks that comes from an energy-intensive process of heating and maintaining the feedwater temperature and inability of MD membrane to handle diverse contaminant mixtures^6,8^. Recently, problem of energy-intensive process has been solved by implementing carbon nanotube/polymer composite as an effective pathway to locally generate the heat at the membrane interface^9^. Yet, the key membrane problem has not been fully addressed till now. Especially, when common chemical or oil-based contaminants are introduced during the MD process, membranes exhibit significant fouling behaviour that rapidly degrades the membrane performance and create irreversible degradation to the membrane^10,11^. Such fouling problem during MD operation reduces water recovery, fails to maintain contaminant rejection, increases the demand for harsh chemical cleaning and rapidly diminishes the lifetime of the membrane. Moreover, conventional MD membrane’s heat conduction across the membrane often leads to low water vapour flux with degradation of performance over a long period of operation that remains as another significant challenge^12^. Such limitations of conventional membranes necessitate new materials for antifouling membranes to successfully address these challenges. Improved MD membranes have been fabricated by several techniques such as phase inversion and electrospinning of polymers. However, most of these methods have been unable to achieve antifouling membranes that demonstrated high water vapour flux and long-term stability during MD operation under diverse mixtures of membrane-damaging contaminants^13,14^.

Despite the small intrinsic pores that restrict the water vapour passage, CVD graphene films possess numerous physiochemical properties that are valuable for MD application. These include its good mechanical strength, thermal and chemical stability, hydrophobicity and atomically thin thickness^15,16^. Recently, enhancements in the performance of water purification processes have been demonstrated with the incorporation of graphene flakes in the membranes^17^. However, until now, the extensive promises and potential of two-dimensional (2D) graphene films for water purification have not been realised.

In this study, we present a low-cost, centimetre-scale CVD graphene membrane with inherent nanochannels to address these challenges. The graphene film is synthesised via a single-step, ambient-air CVD process derived using renewable source such as soybean oil. Graphene film is then directly used as an active layer in MD and exhibits high rates of water vapour flux, excellent salt rejection, long-term stability and sustained antifouling even in the presence of water contaminants (e.g., oil and SDS), which rapidly degrade the performance of conventional commercial MD membranes. In addition, the graphene membrane demonstrates significantly superior performance compared to commercial MD membranes when processing seawater from Sydney Harbour. This performance is achieved without post-synthesis pore generation. Moreover, we show smart way to utilise often disadvantageous feature such as hydrophobicity in CVD graphene as an advantageous feature in emerging desalination and purification process such as MD. Through complementary microscopy, characterisation and computational simulations we propose a new mechanism in water vapour permeation in CVD graphene film through inherent nanochannels of mismatched overlapping grain boundaries.

## Results

### CVD graphene membrane and mechanism of water permeation

**Fig. 1:**
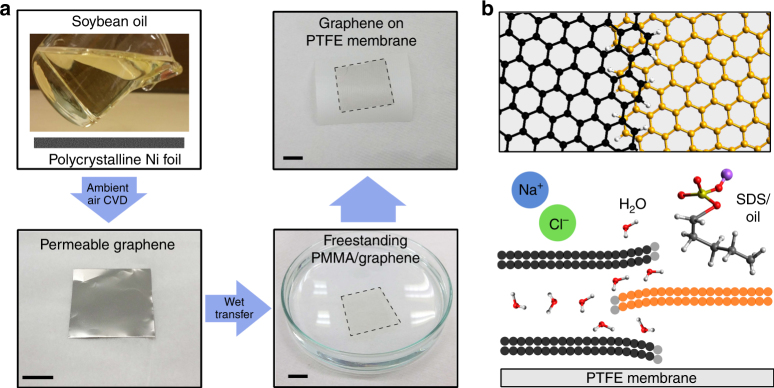
Graphene film synthesis for water desalination membranes. The schematic **a** illustrates the synthesis of permeable graphene using polycrystalline Ni substrate via the ambient-air CVD process using renewable sources such as soybean oil. The synthesised permeable graphene film was transferred to commercial PTFE-based MD membrane for water desalination testing. The proposed mechanism of water purification and desalination is enabled by unique graphene features such as overlapping of graphene domains and grain boundaries **b**, as an advantageous feature in forming antifouling, long-term flux stable MD membrane

The permeable graphene is grown by an ambient-air CVD process, described in more detail elsewhere^18^, and then wet-transferred to a commercial polytetrafluoroethylene (PTFE) MD membrane. This process is described in Fig. 1a. Unlike conventional CVD methods, ambient-air graphene synthesis technique does not require any expensive and explosive purified compressed gases^19,20^. The source for the graphene growth is replaced with a low-cost, safe and renewable bio-source such as soybean oil. The ambient-air CVD process enables the growth of continuous graphene films with a high density of nanocrystalline grain boundaries on polycrystalline Ni substrate, which are desirable as water vapour-permeable channels. Finally, the graphene is wet-transferred onto a conventional supporting commercial PTFE MD membrane. A PMMA-assisted transfer is adopted, and PMMA is removed before testing the graphene-based membrane in water purification (see Supplementary Table 1 for cost analysis)^18^. Figure 1b demonstrates the new proposed mechanism of water permeation in CVD graphene film. Previous studies demonstrate water permeation through pores in CVD graphene that are generated post growth in an energy-intensive, unscaleable process^2,21–23^. In contrast, here we demonstrate that few-to multilayer, nanocrystalline, CVD graphene films with overlapping grain boundaries, which serve as effective water vapour permeation channels, enable a robust antifouling desalination membrane. The membrane can simultaneously reject salt and damaging water-born contaminants such as surfactants and oils.

### Structural properties and features of permeable graphene films

**Fig. 2:**
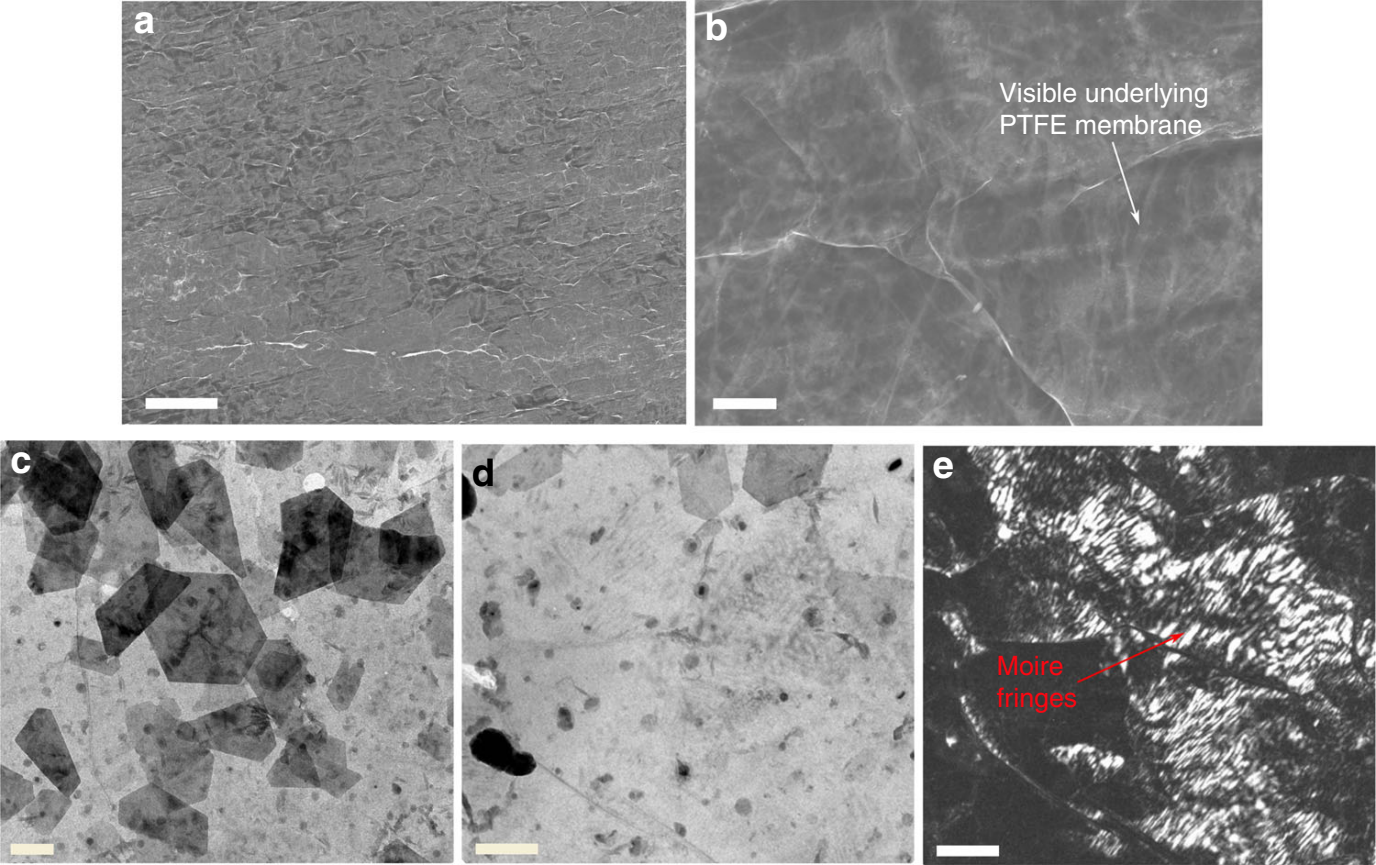
Characteristics of permeable graphene films. Several nanoscopic features in permeable graphene film enable water permeation and desalination. The microscopic morphology of the graphene film was investigated using SEM, revealing the graphene film on the PTFE membrane in **a** low-magnification, scale bar 30 µm and **b** high-magnification, scale bar 2 µm. Many ripples on the surface of the graphene film are observed, and the high transparency of the graphene film allows one to observe the underlying PTFE membrane. TEM images in **c** low-magnification, and respective **d** bright-field and **e** dark-field images show many small graphene domains with many thick dark lines corresponding to the overlapping of grain boundaries that form the channels for the water vapour passage. **c–e** Scale bars represent 200 nm

The morphology and structural properties of the graphene film is analysed by scanning electron microscopy (SEM) and transmission electron microscopy (TEM; Fig. 2 and Supplementary Fig. 1). The transferred graphene film homogeneously coated the PTFE membrane as is evident from SEM images taken at low and high magnifications (Figs. 2a, b). The graphene film is shown to conform to the membrane surface, as suggested by the visible wrinkles in the graphene film over the partially visible underlying membrane. Furthermore, the distribution of domain sizes, domain orientations and thickness within the graphene film were characterised. A continuous few-layer graphene film with randomly oriented, overlapping stacked graphene layers, which often show hexagonal morphology indicative of single crystallinity, is identified in low-magnification TEM (Fig. 2c). Bright-field (Fig. 2d) and dark-field (Fig. 2e) TEM imaging demonstrates that the base few-layer graphene is polycrystalline with mis-oriented domains, ranging from ~200 to 600 nm, indicated by the variations in contrast at the domain boundaries and the presence of Moire fringes (periodic stripes) within the graphene domains.

Importantly, we observe a slight overlap of domain boundaries in multiple regions of the sample—these serve as potential channels for the passage of water molecules (Supplementary Fig. 2a-b)^24^. On the basis of TEM image contrast these channels are ~10 nm overlapping and extend along the length of the grain boundaries ~400 nm–1 µm. Channel height is the interlayer spacing of graphene film; specifically, for turbostratic CVD graphene layers grown on nickel as in our experiment, this value is 0.34 nm^25^. High-resolution TEM and selected area electron diffraction (SAED) analysis of these nanochannels is constrained because of the multilayer thickness and small channel width compared to selected area aperture size.

**Fig. 3:**
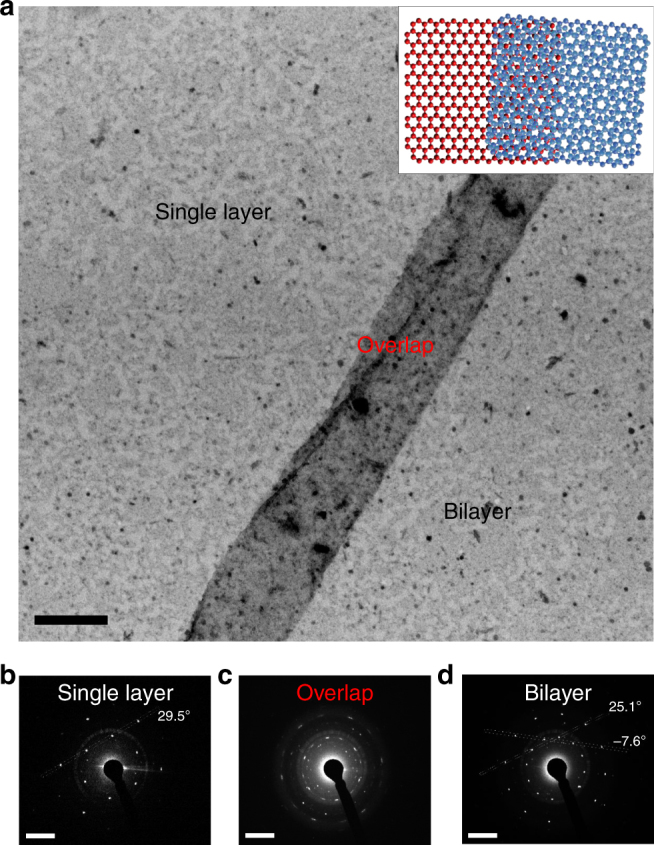
TEM characterisation of overlapping domains forming nanochannel in graphene film. **a** TEM image of overlapping domain boundary (darker contrast region) forming extended nanochannel in permeable graphene film; scale bar is 200 nm. SAED patterns (and associated line profiles in Supplementary Information) confirm the labeled regions as **b** single-layer graphene with rotation axis of 29.5°, **c** overlapped domains forming ~250 nm wide nanochannel, **d** turbostratic bilayer graphene with rotation axes of −7.6° and 25.1°. **b–d** Scale bars are 5 nm^−1^. The darker contrast region is confirmed as an overlapping mis-oriented graphene domain boundary, or nanochannel, due to the single layer to bilayer transition and shift in the respective rotation axes on either side of the feature. Inset shows representative diagram of an overlapping domain boundary with equivalent rotations of domains but narrow nanochannel width

To provide conclusive evidence for the presence of the nanochannels in the permeable graphene, samples of predominately single or bilayer graphene with wider nanochannels were synthesised. Darker contrasted nanochannels with varying channel length >1 µm and varying channel width >100 nm are visible (Fig. 3a and Supplementary Fig. 4, Supplementary Fig. 5). SAED of the wider nanochannels is possible and confirms the presence of overlapping domain boundaries rather than folds or wrinkles of the graphene film. In particular, Fig. 3 demonstrates an ~250 nm wide nanochannel formed over 2.5 µm length due to the mis-oriented overlap of single-layer region (Fig. 3a, left side) and a turbostratic bilayer region (Fig. 3a, right side) regions. The darker contrast region is confirmed as an overlapping mis-oriented graphene domain boundary (or nanochannel) due to the single layer to bilayer transition and shift in the respective rotation axes on either side of the feature (29.5° on single-layer side (Fig. 3b), and −7.6° and 25.1° on turbostratic bilayer side (Fig. 3d; see inset of Fig. 3a for a representative diagram showing relative rotations of domains). It is worth noting that while the existence of nanochannels could be confirmed using predominately single or bilayer graphene film grown from ambient-air CVD process. However, these samples are fragile and inferior membranes compared to the few-to-multilayer graphene. The unique morphology of the permeable few-layer graphene film features high-density of submicrometre polycrystalline grains with numerous grain boundaries. The overlapped and mismatched graphene boundaries produce nanochannels, thus opening multiple passages for the efficient transport of water vapour.

**Fig. 4:**
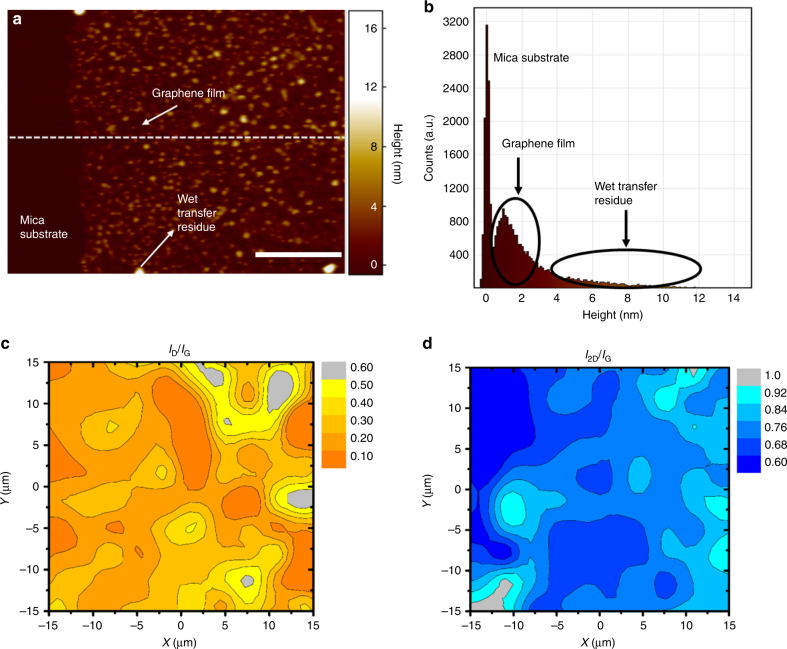
Additional structural features of permeable graphene films. Additional characterisation of the graphene films reveals their rough surface texture and variation in thickness. These features are favourable for generating a bottleneck region for water vapour permeation. The presence of multiple nanocrystalline domains suggests the existence of numerous channels for water vapour permeation. **a** An AFM topography image of the edge of a graphene film deposited on a mica substrate; the dark region at the left hand side of the image is the mica substrate; the lighter region is the graphene film; and the bright spots are most likely residue from the wet-transfer process; scale bar is 1 µm. **b** A relative height histogram of the AFM image (**a**). The high narrow peak around 0 nm height represents the mica substrate, the broader distribution represents the graphene film and the tail up to heights of 18 nm most likely represents wet-transfer process residue. **c**, **d** Raman spectral mapping analyses of the intensity ratios of *I*_D_*/I*_G_ and *I*_2D_*/I*_G_

The structural properties of the graphene film were further examined by Raman spectroscopy mapping and atomic force microscopy (AFM; Fig. 4). The multilayer graphene film covers the entire surface, with regions of varying thickness. AFM topography imaging of the graphene film shows a thickness ranging from 0.7 to 3.7 nm (~2–10 graphene layers), and a mean film thickness of 1.7 nm (Figs. 4a, b). The wet-transfer process likely produces contaminants (e.g., PMMA residue and Fe particles) on the surface of graphene. Furthermore, transmittance of the graphene film is measured to examine the average film thickness. A transmittance of 85% at 550 nm is observed (Supplementary Fig. 6). Raman characterisation indicates that the graphene is a few-layer polycrystalline film. Raman spectral mapping of *I*_D_*/I*_G_ and *I*_2D_*/I*_G_ intensity ratios (which shows defect and relative thickness distribution) is conducted to determine the defect and the thickness uniformity in the films (Figs. 4c, d and Supplementary Fig. 7). Three distinct peaks are present in the Raman spectra of graphene, namely, the characteristic disorder peak that arise from defects in the *sp*^2^ carbon (D-band) at ~1350 cm^−1^, the graphitic peak that arise from the in-plane vibrational E_2g_ mode of the *sp*^2^ carbon (G-band) at ~1580 cm^−1^, and the second-order 2D-band that arise from three-dimensional inter-planar stacking of hexagonal carbon network at ~2670 cm^−1^ ^26^. The intensity ratios of *I*_D_*/I*_G_ is 0.1–0.3 and that of *I*_2D_*/I*_G_ is 0.6–1 (Figs. 4c, d). This disorder content may be attributed to defects, which arise from grain boundary interactions by analysing the G peak. The *I*_2D_*/I*_G_ intensity ratio suggest that the film is composed of few-layer graphene, with variations in film thickness from 2 to 10 atomic layers. These characterisations are in a good agreement with TEM and other characterisations.

### Graphene for desalination of water with fouling contaminants

The performance of the permeable graphene-based (graphene/PTFE-based MD membrane) membrane was carried out by direct contact MD (DCMD) using a range of solution mixtures including highly saline solutions with the presence of surfactants, mineral oil and real seawater collected from Sydney Harbour. The water vapour flux and salt rejection were measured to characterise the purification of water by the membranes. Performance of the permeable graphene-based membrane is benchmarked against the commercial PTFE MD membrane (Ningbo Changqi, 120 µm thickness, 0.4 µm pore size). The testing is carried out in a continuous, co-current crossflow system illustrated in Supplementary Fig. 8.

**Fig. 5:**
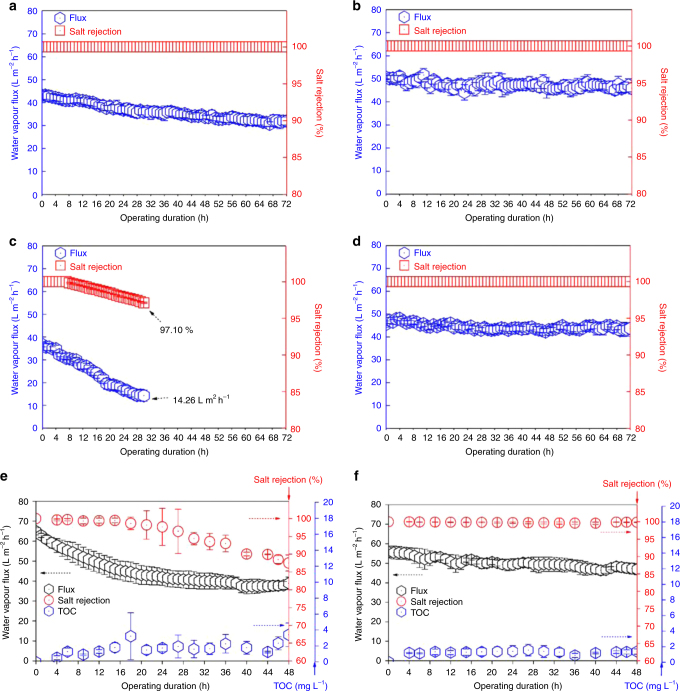
Desalination performance of commercial and permeable graphene-based MD membranes. Water vapour flux and salt rejection performances of the membranes: **a** commercial PTFE-based MD membrane and **b** permeable graphene-based membrane in the DCMD process for 72 h, with 70 g L^−1^ of NaCl solution as feed. **c** Commercial PTFE-based MD membrane and **d** permeable graphene-based membrane with 70 g L^−1^ NaCl solution and 1 mM sodium dodecyl sulphate (SDS) as feed. The flow rates of these DCMD tests were both maintained at 6 L h^−1^ in the feed and permeate stream. **e** Commercial PTFE-based MD membrane and **f** permeable graphene-based membrane in the DCMD process with a feed solution containing 1 g L^−1^ mineral oil with 70 g L^−1^ of NaCl and 1 mM NaHCO_3_ in the DCMD process for 48 h. The feed and permeate temperatures were 60 and 20 °C, respectively. The flow rates of these DCMD tests were both maintained at 30 L h^−1^ in the feed and permeate stream. The error bar represented SD from three repeated filtration tests. Our results demonstrate that the permeable CVD graphene-based membranes exhibit strong antifouling properties while enabling rapid water vapour permeation and good salt rejection

With NaCl feed solution (70 gL^−1^ NaCl, typical concentration of brine water), both the permeable graphene-based membrane and commercial PTFE membranes exhibit similar salt rejection, 99.9% after 72 h of operation. A relatively higher water vapour flux was observed for the permeable graphene-based membrane in comparison with the pristine PTFE-based MD membrane (Figs. 5a, b, Supplementary Fig. 9a-b and Supplementary Fig. 10a-b). Moreover, to explore the water vapour permeation-governing factors in permeable graphene-based membrane, we tested the membrane under different crossflow rate of the water streams and under different temperature gradient created by increasing the temperature of the feedwater (Supplementary Fig. 11). The result shows that as the crossflow rate of the water streams increased, we observed a systematic increase in the water vapour flux (Supplementary Fig. 11a). Similarly, as the temperature gradient increases, we observed a systematic increase in the water vapour flux (Supplementary Fig. 11b). These observations reveal that the crossflow rate and the temperature gradient are important controls of water vapour permeation through the graphene. Importantly, in both cases, at different cross flows and under different temperature gradients, the permeable graphene-based membrane exhibited stable water vapour flux over the total duration of MD operations.

In MD separation processes, liquids with low surface tension (i.e., saline solution with surfactant such as sodium dodecyl sulphate (SDS)) cause detrimental pore fouling and/or membrane wetting, thus leading to inferior performance (see Supplementary Fig. 12)^27,28^. Therefore, to explore the permeable graphene-based membrane’s performance in solutions containing fouling materials, we tested the pristine PTFE and permeable graphene-based membranes using saline solutions containing surfactants such as SDS. As expected, significant fouling is evident for the pristine PTFE-based MD membrane. This membrane is able to process saline/SDS feed solution (70 g L^−1^ NaCl with 1 mM SDS), but with a significant reduction in water flux from 40 L m^−2 ^h^−1^ to 14.2 L m^−2^ h^−1^ over 32 h (see Fig. 5c and Supplementary Fig. 9c-d). In addition, salt rejection decreases significantly, from 100 to 97.1%. In contrast, the permeable graphene-based membrane demonstrated stable and high water vapour flux (50 L m^−2^ h^−1^) and stable salt rejection (100%) over 72 h of MD operation under similar operation conditions (see Fig. 5d and Supplementary Fig. 10c-d).

The permeable graphene-based membrane is then also tested with the inclusion of high concentration of oil compounds—another common contaminant that causes significant wetting and fouling problems in widely used MD membranes such as commercially available PTFE and PVDF-based MD membranes (see Figs. 5e, f and Supplementary Fig. 12). Substantial fouling is evident for the pristine PTFE-based MD membrane when processing the saline water/mineral oil (see Supplementary Fig. 13a-b for the emulsion mixtures used for experiment and its oil size distribution and Supplementary Table 2) feed solution (1 g L^−1^ mineral oil with 70 g L^−1^ of NaCl and 1 mM NaHCO_3_; Fig. 5e). This is indicated by a continuous decrease of water flux from 62 to 38 L m^−2^ h^−1^, and a significant reduction in the membrane’s salt rejection capacity from 100 to 87% over 48 h.

In contrast, the permeable graphene-based membrane outperformed the commercial PTFE-based MD membrane, demonstrating a significant improvement in the salt rejection (100 to 99.9%) and retention of water vapour flux (54 to 49 L m^−2^ h^−1^) for the 48 h of MD operation under similar conditions (Fig. 5f). Although significant oil mixtures are visible on the graphene surface after the MD operation (see Supplementary Fig. 13c), our result indicates that wetting or fouling of the membrane surface was insignificant in the permeable graphene-based membrane, unlike the commercial PTFE-based MD membrane case.

Moreover, all the experiments were repeated to demonstrate the reproducible performance (see Supplementary Fig. 9 and Supplementary Fig. 10). During our repeat experiments, we also monitored the total organic carbon (TOC) level in the permeated water streams (to examine oil rejection) over 48 h (see Supplementary Fig. 9e-f and Supplementary Fig. 10e-f). The results show that stable organic carbon and oil rejection was achieved over 48 h of MD operation through the permeable graphene-based membrane, while the PTFE-based MD membrane exhibited poor oil rejection evidenced by a higher TOC level over 48 h. Furthermore, all the experiments (see Supplementary Fig. 9 and Supplementary Fig. 10) reveal that the permeable graphene-based membrane has significant advantages over the commercial membrane. Indeed, the graphene membrane consistently demonstrates stable, reproducible, antifouling performance with excellent salt and oil rejection, especially in saline water containing fouling contaminants such as surfactants and oils where the conventional membrane fails.

### Desalination of seawater from Sydney Harbour

**Fig. 6:**
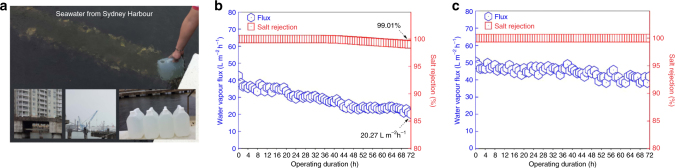
Desalination of seawater from Sydney Harbour. **a** Photographs of the water extraction process and site. **b**, **c** Water vapour flux and salt rejection performances of **b** a commercial PTFE-based MD membrane and **c** the permeable graphene-based membrane in the DCMD process for 72 h. The feed and permeate temperatures were 60 and 20 °C, respectively. The flow rates of all DCMD tests were both maintained at 30 L h^−1^ in the feed and permeate stream. Strong antifouling nature of permeable graphene film with high and stable water vapour flux are demonstrated over a long operation time. Stable, 100% salt rejection rate is maintained

To demonstrate practical applicability of the permeable graphene-based membrane under real desalination conditions, we carried out water desalination tests using unprocessed real seawater feed (total dissolved solids of 34.2 g L^−1^; Fig. 6). Real seawater is collected from the Sydney Harbour, NSW, Australia (see Supplementary Table 3 showing the composition of real seawater). The seawater collection site is central to an environment of households and ongoing industrial activities (see Supplementary Fig. 14). The commercial PTFE-based MD membrane fouled while processing seawater showing a continuous reduction in water vapour flux (40–20 L m^−2^ h^−1^) over 72 h, and a slight decrease in salt rejection (100–99%; Fig. 6a).

Conversely, the permeable graphene-based membrane exhibited superior performance in salt rejection (100%), while maintaining a high water vapour flux (50 to 46 L m^−2^ h^−1^) and long-term stability over 72 h (Fig. 6b). The permeable graphene-based membrane with the area of 4 cm^2^ was able to process 0.4~0.5 L of real seawater per day without degradation. Moreover, to demonstrate the permeable graphene-based membrane’s long-term stability under real seawater feed, we have tested the membrane performance for prolonged duration (120 h) of MD operation (see Supplementary Fig. 15). Over 120 h of MD operation, we observed a stable water vapour flux with excellent salt rejection of 99.99%, thus demonstrating the membrane’s excellent long-term stability. Furthermore, the concentration polarisation effect was insignificant even during the prolonged processing of real seawater containing multiple components. Overall, our results demonstrate that our ambient-air-derived CVD graphene films are promising active materials for MD. MD appears to be one of the promising applications where hydrophobic CVD graphene films can be applied in water purification to help solve some of the persistent membrane-related issues in a broader water purification context.

### Mechanism of water permeation

**Fig. 7:**
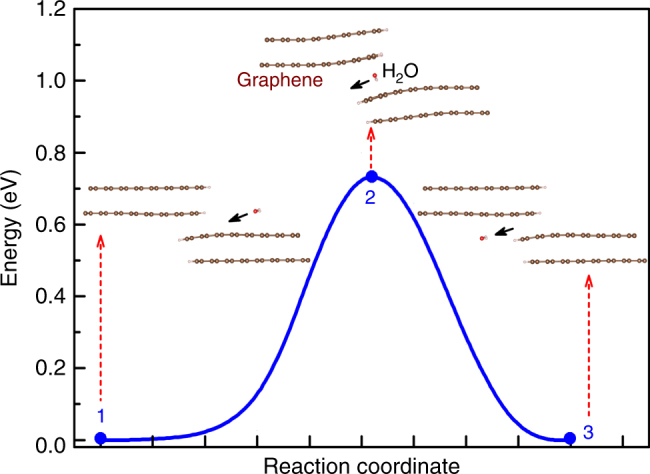
Water permeation energy required for water vapour passage through overlapping grain boundaries in graphene. The minimum energy pathways of the H_2_O migration through the channels created by two overlapping grain boundaries in multilayer graphene sheets. Simulation results show that sufficient energy needs to be supplied to the graphene film in order for water vapour permeation to occur through the graphene channels, which is supplied in the form of heat in the desalination process

To visualise the water vapour permeation through our graphene structures, numerical simulations are carried out to explain the water permeation and the kinetics of water passage through overlapping graphene grain boundaries. Two simulation methodologies were employed. The first simulation is based on first-principles (ab initio) solid nudged elastic band method, which is used to evaluate the energy barrier of water migration through graphene layers (i.e., bottleneck region). The model is simplified by simulating one water molecule penetrating through the graphene layers as shown in Fig. 7. It can be clearly seen that the water molecule causes the bending of sheets away from the H_2_O because of the hydrophobic nature of graphene. In addition, the calculated energy barrier during the whole process is ~*E* = 0.78 eV, which is a moderate value and can be overcome kinetically. In our experiments, kinetic energy is applied in a form of heat where the temperature difference between the hot feed and cold permeate will supply enough kinetic energy to overcome this water vapour permeation barrier. It is also expected that this energy barrier can be further reduced when more water molecules (higher water flow) are introduced, suggesting that the proposed water transport process is feasible (See Supplementary Movie 1).

**Fig. 8:**
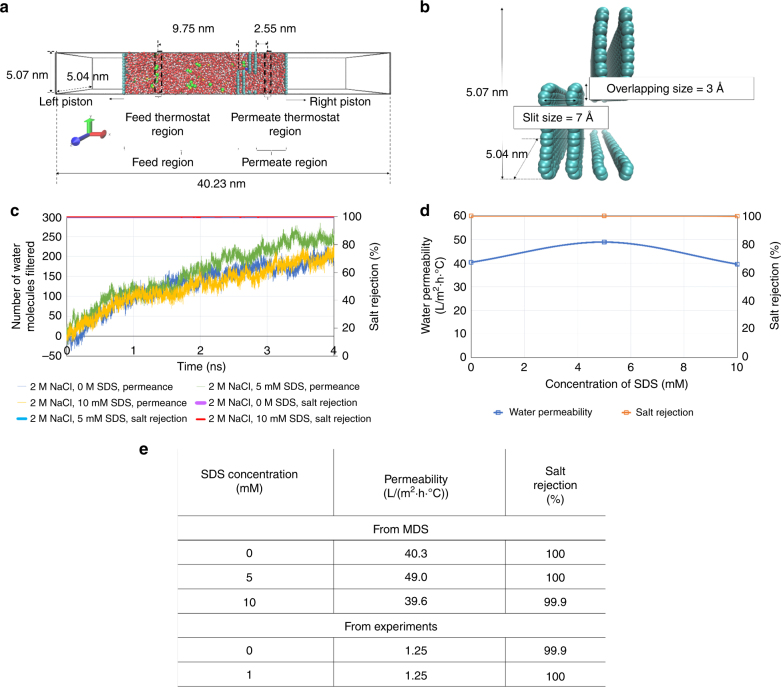
Molecular dynamics simulation of water permeation through overlapping grain boundaries in graphene. The schematic (**a**) describes the simulation domain set up for the molecular dynamics study. **b** The molecular construct of the permeable graphene-based membrane, deduced from the characterisations (SEM, TEM, Raman spectroscopy and AFM) carried out on the membrane sample used for experimental testing. **c** The water permeation and salt rejection time history recorded during the molecular dynamics simulations. **d** The variation of water permeability and salt rejection with increasing concentration of SDS. **e** Comparison of numerical values of permeability obtained from MDS and from experiments

The second computational study is based on molecular dynamics simulation (MDS). MDS resolves molecular length scales, and therefore provides a molecular picture of the transport of water molecules and rejection of salt ions through the graphene layers. The simulation domain and molecular construct of the permeable graphene-based membrane are shown in Figs. 8a, b. Three feed solutions are considered in this study: water with 120 g L^−1^ of sodium chloride (NaCl), water with 120 g L^−1^ of NaCl and one molecule (5 mM) of SDS, and finally water with 120 g L^−1^ of NaCl and two molecules (10 mM) of SDS. Higher concentrations of NaCl are used in the simulations as compared to the experiments to ensure sufficient interactions of the salt ions with the membrane, given the very short simulation time of 4 ns. Here, one or two SDS molecules are added in the feed, as the experimental scenario of 1 mM of SDS corresponds to either a single- or bi-molecular state of SDS^29^.

The dynamics of water molecules, NaCl and SDS ions can be observed in Supplementary Movie 2-3, while the water permeation and salt rejection behaviour are quantified in Figs. 8c–e. Similar to the experimental observations, MDS results also reveal a constant water flux across the graphene membrane for all three feed solutions, as evident from the linear water permeance graphs in Fig. 8c. In addition, Fig. 8c also shows that the salt rejection remains close to 100% for all three feed solution configurations, which agrees well with the experimental results. Moreover, MDS results show that permeability remains high at higher SDS concentrations (Fig. 8d), consistent with the experimental observations. The numerical permeability values derived from MDS are about an order of magnitude higher than the experimental results. This is probably due to the idealised conditions in the MDS, and the simulation model does not take the PTFE support layer into account. Overall, the MDS results further support the proposed water transport mechanisms through the overlapping grains of graphene. Further details of the simulations can be found in Supplementary Fig. 16 and Supplementary Fig. 17 and Supplementary Table 4.

## Discussion

Unlike the previous studies where post-treatment techniques create nanopores in the graphene surface, we did not observe nanopores in our graphene microstructures. Rather we observed a multilayer graphene film with numerous graphene grain boundaries arising from the small domain sizes and numerous overlapping regions of adjacent graphene grains with the mismatched graphene grain boundaries.

These nanochannels created by mismatched and overlapping graphene domains would facilitate fast transport of water vapour^30^. This is possible because flow resistance is much reduced when water or water vapour is transported between graphene sheets^31^. Multiple characterisations (i.e., AFM, SEM and TEM) show that our graphene film had variations in the number of layers over microscopic regions induced by mis-oriented, overlapping and sub-micron-sized grains. These structural features promote deformations (i.e., wrinkling) in graphene films^30,32^. Such nanoscopic wrinkling would increase the surface roughness of our graphene films and create suitable surface structures (i.e., nanoscopic bottleneck sites) to promote the water vapour entry and rapid permeation (Supplementary Fig. 18).

Another aspect of relevance to the MD process is heat conduction, which should be reduced across high-performance MD membranes^33^. To test the hypothesis that our permeable graphene might contribute to the reduction of heat conduction across the graphene-enhanced PTFE membrane as compared to the pristine PTFE membrane, we carried additional vapour permeation experiments under high temperature gradient (*ΔT* = 70 °C) conditions, which is 30 °C higher than elsewhere in this work. We placed a temperature probe as close as possible to each (feed and permeate) side of the membranes to measure the actual temperature difference across the both membranes as the vapour permeates. The results in Supplementary Fig. 19 show that under such conditions the commercial PTFE MD membrane failed as the water vapour flux increased rapidly over the short duration of the MD process. On the contrary, the permeable graphene-based membrane exhibited consistently stable water vapour flux (Supplementary Fig. 19a). Moreover, the permeable graphene-based membrane was able to maintain stable and slightly higher actual temperature gradient compared to the pristine PTFE membrane (Supplementary Fig. 19b).

These results suggest potential additional advantages of using permeable graphene-based membranes in MD process under higher thermal load conditions. These findings are consistent with the fact that graphene is a two-dimensional nanomaterial with high anisotropy in thermal conductivity, where high thermal conductivity is observed in the *x–y* direction due to *sp*^2^ bonding in graphene lattice and poor thermal conduction in the *z* direction arising from weak van der Waals interaction^16,17,34,35^. This obviously favourable feature for MD applications requires further experimental and theoretical studies in near future.

We have also studied the effect of graphene on the mechanical strength of the membrane. The results show only a marginal improvement compared to the pristine PTFE membrane (see Supplementary Fig. 20). Because of the small thickness of the permeable graphene film, the improvement was not significant compared to the cases when a large quantity of graphene or carbon nanotubes is blended with the polymer membrane material^36,37^.

Surface energy plays a critical role in antifouling and antiwetting properties of the MD membrane. The ideal MD membrane should be hydrophobic with a high water contact angle. While our graphene-based membranes are mildly hydrophobic (contact angle of 81.3°), they show better antifouling and antiwetting performance compared to the highly hydrophobic surface of commercial PTFE-based MD membranes with the contact angle of 131.3° (see Supplementary Fig. 21).

Some other factors could also reduce the blocking of the water passage channels by the contaminant molecules. To understand the antifouling nature of our permeable graphene film, we calculated the adsorption energy to investigate the interactions between the contaminant particles such as SDS with nanochannels at grain boundaries. The calculated adsorption energy *E*_ad_, of one SDS molecule at the grain boundary is −2.36 eV, while the adsorption energy of H_2_O is −0.12 eV. Therefore, the interactions between the graphene and the contaminant molecules are of the weak physisorption type. Similar values of the adsorption energy are expected for molecules with a similar chemical structure as of SDS (e.g., mineral oil). The weak physisorption of the contaminant molecules on the graphene surface is overcome because of the kinetic energy provided by continuous feedwater flow.

To experimentally verify the weak physisorption interaction between SDS and the graphene surface, we have repeated the experiments with the pristine PTFE and the permeable graphene-based membranes with SDS/saline water mixtures for 72 h, and then samples were dried without any cleaning process and analysed using Raman spectroscopy. Knowing that SDS has clear and distinct Raman peaks, the areal mapping of SDS Raman intensities was then carried out to find out the relative difference in SDS adsorption on the pristine PTFE and permeable graphene membrane surfaces (see Supplementary Fig. 22). The results show the much lower SDS Raman intensities in the latter case, thus evidencing weaker SDS interactions with the graphene surface compared to pristine PTFE membrane; this validates the adsorption energy calculations.

To further investigate the antifouling properties of permeable graphene, we have carried out the surface energy calculations and the zeta-potential measurements. The membrane’s surface energy and charge compared to the pristine PTFE membrane are summarised in Supplementary Table 5 and Supplementary Fig. 23. Although the surface energy is higher (lower contact angle) compared to the PTFE membrane, the permeable graphene film exhibits a negligible surface charge (remains charge neutral) compared to the negatively charged PTFE membrane. Charge neutrality generally helps improve the membrane’s antifouling properties, and is likely to be the case in this work^38,39^.

MDS provide additional insights into the antifouling effects of the graphene-based membrane against contaminant molecules. In the simulations, the SDS molecules were initially placed near the nanochannel, and then remained close to the nanochannel because of the weak physisorption (Supplementary Fig. 17 and Supplementary Fig. 24). The MDS results in Fig. 8 indicate that the permeability did not decrease in the presence of SDS contaminant. By visualising the simulations (see Supplementary Fig. 24), we observed that when SDS molecules stay near the graphene membrane, the number of salt molecules near the channel entrance is much reduced. This makes it easier for the water molecules to pass through the channel. This is another factor to keep the permeability stable in the presence of SDS contaminant molecules.

Utilisation of CVD graphene in water purification has remained limited in both research and industrial scales because of difficulties in precise pore engineering, high production costs and the hydrophobic nature of graphene films. In this work, the above challenges have been addressed by the low-cost, green (i.e., renewable natural precursors) and resource-efficient (i.e., no purified gases or extensive vacuum processing) synthesis of permeable graphene films. Importantly, the synthesised graphene films possess a high density of nanochannels that are distributed across the submicrometre domains. Without any post-synthesis pore engineering, the permeable graphene films demonstrate high-water flux (~50 Lm^−2^h^−1^ for 4 cm^2^, up to ~0.5 L per day) excellent salt rejection (99.9%) when processing highly saline water (i.e., NaCl of 70 g L^−1^), and exceptional antifouling properties by rejecting common water-born contaminants (i.e., oil and surfactants) over a prolonged period (48 and 72 h) of MD operation. Notably, we have also demonstrated the long-term, effective desalination of seawater, directly sourced from Sydney Harbour. Synergistic integration of our graphene films provided solutions to membrane problems persisted in MD as well as exhibiting significant advantages over widely used commercial MD membranes (e.g., PTFE and PVDF). The outcomes of this work may near commercial applications of CVD graphene films in water purification, and also demonstrate the benefits of using 2D materials as advanced membranes for water purification and desalination.

## Methods

### Compressed gas-free, ambient-air CVD of permeable graphene

The growth of graphene was carried out in a thermal CVD furnace with a quartz tube. Polycrystalline Ni foils (25 µm, 99%, Alfa Aesar) were used as the growth substrate. The experimental schematic is shown in Fig. 1. Briefly, two alumina plates were placed in the heating zone of the furnace. One alumina plate was loaded with 0.17 mL of soybean oil precursor, and the other was loaded with the Ni foil growth substrate. The openings of the quartz tube were then sealed. The growth of graphene proceeds with a gradual heating and fast quenching temperature profile. Firstly, the furnace temperature was raised to 800 °C. This was followed by holding at 800 °C for 3 mins. After the growth step, the chamber was evacuated, and the sample remained in the heating zone for some time. The sample was then rapidly removed from the heating zone to be cooled to ensure the homogeneous and continuous graphene films to segregate. Because of the evaporation and thermal expansion of the precursor material, a small build-up in pressure within the tube was observed. Throughout the heating stage (200–800 °C), atmospheric pressure was maintained in the quartz tube by allowing this build-up of gases to exit via the exhaust of the tube. A controlled gas environment was created in the tube by circulating the gases produced by precursor evaporation. Following the heating stage, pressure within the quartz tube was stabilised at atmospheric pressure. No additional gases were introduced into the quartz tube throughout the entire growth process. Such growth process resulted in the formation of polycrystalline, few-to multilayer graphene sheets with numerous grain boundaries. For the further TEM analysis (to confirm the existence of nanochannels through the overlap of graphene domain boundaries), a thinner graphene film (predominantly single to bilayer) was synthesised by using the lower precursor amount of 0.155 mL while keeping the other protocols the same.

### Transfer of graphene

A poly (methyl methacrylate) (PMMA)-assisted transfer of graphene was adopted. Briefly, 46 mg/mL of PMMA (*M*_w_ 996,000 Sigma-Aldrich) was spin-coated onto the as-grown graphene on Ni foil (3000 rpm for 1 min). The sample was then dried in open air for 12 h. Subsequently, the underlying Ni foil was dissolved in 1 M FeCl_3_ in 30 min. The PMMA/graphene film then floated to the surface. This was washed several times with deionized (DI) water. Next, the PMMA/graphene was lifted off from the DI water bath and transferred onto the membrane substrate. The PMMA was then dissolved with acetone, and the sample is rinsed with DI water.

### Microscopy and microanalysis

Raman spectroscopy was performed using a Renishaw inVia spectrometer with Ar laser excitation at 514 nm and a probing spot size of ~1 µm^2^. AFM images were acquired with an Asylum Research MFP-3D AFM operating in intermittent contact (tapping) mode with a Budget Sensors TAP150Al-G cantilever (DESCRIBE THESE PARAMETERS *f*_*R*_ = 123 kHz, *Q* = 1745 and *k* = 2.1 Nm^−1^, with free-air amplitude = 100 nm and feedback set-point = 70%). Image analysis was performed using the Scanning Probe Image Processor (SPIP^TM^) software produced by Image Metrology A/S. Energy-filtered TEM was performed using a JEOL 2200FS TEM microscope operated at 200 kV. Optical images were obtained with an Olympus BX51 optical microscope. Transmittance measurements were obtained using a Varian Cary 5000 UV–Vis spectrophotometer. A graphene area of 2 cm^2^ was used, and optical spectra were recorded in the wavelength range from 300 to 800 nm.

### Membrane distillation set-up

DCMD was conducted using a closed-loop bench-scale membrane test apparatus (see Supplementary Fig. 8). The membrane cell was made of acrylic plastic to minimise heat loss to the surrounding. The flow channels were made in each of two acrylic blocks that constitute the feed and permeate semi-cells. Each channel is 0.3 cm deep, 2 cm wide and 2 cm long; and the total active membrane area was 4 cm^2^. Temperatures of feed and distillate solutions were controlled by two heater/chillers (Polyscience, IL, USA), and were continuously recorded by temperature sensors that were inserted at the inlet and outlet of the membrane cell. Both feed and distillate streams were concurrently circulated by two gear pumps. The same crossflow rate of 30 L h^−1^ (corresponding to the crossflow velocity of 9 cm s^−1^) was applied to both feed and distillate simultaneously in order to minimise the pressure difference across the MD membrane. Weight change of the distillate tank was recorded by an electronic balance (Mettler Toledo, OH, USA) with a data logger. All piping used in the DCMD test unit was covered with insulation foam to minimise heat loss.

### Experimental protocol for MD using saline water

MD fouling experiments were conducted using four types of saline water and saline water with contaminant mixtures: 70 g L^−1^ NaCl solution, 70 g L^−1^ NaCl solution with 1 mM SDS, 70 g L^−1^ NaCl solution with 1 g L^−1^ mineral oil and 1 mM NaHCO_3_ (the oil emulsion was prepared by vigorous mixing using modular homogenisers at a speed of 20,000 rpm for 30 min) and real seawater, respectively. The comparison between the mineral oil and light crude oil is tabulated in Supplementary Table 2.

Feed and distillate volumes of 4 and 1 L were used, respectively. The temperature of inlet feed solution is 60 °C, while that of the distillate inlet stream is 20 °C in most of the experiments. In the high temperature gradient (*ΔT* = 70 °C) measurements, the feed solution temperature was 90 °C, while the distillate temperature was also 20 °C. To measure the actual temperature difference across the membranes as the vapour permeates, a temperature probe was placed as close as possible to each (feed and permeate) side of the membranes. A new membrane sample was used for each experiment. Water vapour flux was recorded with a digital balance continuously. Conductivity of the distillate was measured by a conductivity meter (HQ14d, Hach, CO) every 5 min. All feed solutions were processed by DCMD for 72 h, except for the cases of mineral oil tests that was performed for 48 h and the case where we demonstrate long-term stability with real sea water feed, which was performed for 120 h. TOC was analysed using a TOC/TN analyser (TOC-VCSH, Shimadzu, Kyoto). Membrane surface charge was measured by a SurPASS electrokinetic analyser (Anton Paar CmbH, Graz, Austria). Zeta potential of the membrane surface was calculated from the measured streaming potential using the Fairbrother-Maastin approach. All streaming potential measurements were performed in a background electrolyte solution (i.e., 10 mM KCl). The background solution was also used to completely flush the cell before pH titration using either hydrochloric acid (0.5 M) or potassium hydroxide (0.5 M). Surface energy of the membranes were calculated by measuring the contact angle using three different liquids (two polar and one non-polar) of well-known surface tension, employing the Lifshitz van der Walls (non-polar) and Lewis acid–base (polar) approaches^40^. Commercial PVDF MD membrane (Durapore, 0.45 µm pore size, 280 µm thickness) experiments were carried out in same experimental protocol as PTFE membrane case.

### Numerical simulations

First-principles calculations were performed based on the density functional theory (DFT) using the plane-wave basis VASP code^41,42^. The generalised gradient approximation in the Perdew, Burke and Ernzerhof form (GGA-PBE) is used as exchange correlation functional^43^. A damped van der Waals correction was incorporated using Grimme’s scheme^44^ to better describe the non-bonding interaction. The projector-augmented-wave method^45^ was used to describe the electron−ion interaction and the plane-wave energy cutoff was set to 400 eV. A vacuum layer with a thickness of at least 15 Å was used. Atoms were relaxed until the residual force and energy converged to 0.01 eV/Å and 5 × 10^−5^ eV, respectively. A *Γ*-centred *k*-point mesh^46^ of 1 × 1 × 1 was used for sampling in the first Brillouin zone. The energy barrier for H_2_O migration through the graphene sheets was obtained by using the climbing image-nudged elastic band (CI-NEB) method^47^. For the graphene grain boundaries, the adsorption energy *E*_ad_ of the SDS and H_2_O molecule is obtained according to

$$E_{\mathrm{ad}} = E_{\mathrm{comb}} - E_{\mathrm{bound}} - E_{\mathrm{mo}}$$

where *E*_comb_*, E*_bound_ and *E*_mo_ represent the total energy of the SDS molecule/H_2_O-adsorbed graphene boundary, pure graphene boundary and the SDS or water molecule, respectively.

The large-scale atomic/molecular massively parallel simulator^48^ is used for the MDS computations and visual molecular dynamics (VMD)^49^ is used to visualise the computed results. The boundary conditions are periodic in all three dimensions, with a simulation cell depicted in Fig. 8a. Sufficient pressure is applied on both left and right pistons to keep the density of water ~1 g/cm^3^, and the pistons are allowed to move in the *x* direction under zero differential pressure. The membrane is constructed from four graphene sheets with a slit size between each sheets of 7 Å, and an overlapping size of 3 Å, as shown in Fig. 8b. For the force models, the TIP4P water model with a long-range Coulombic particle–particle particle–mesh solver is used to model the interactions between water molecules in our simulation^50^. For the SDS molecules, the force field described by Tummala and Striolo is employed in our study^51^. The Lennard–Jones (L–J) potentials for the sodium ions, chlorine ions and carbon atoms are adapted^51^. The intermolecular L–J interactions are then computed using the Lorentz–Berthelot mixing rule. In this study, the membrane carbon atoms are kept fixed in position. A more detailed description of the force potentials used can be found in Supplementary Table 4. The system is subjected to equilibration for 1 ns, before the actual data collection run begins where the feed solution is maintained at a temperature of 345 K with a Langevin thermostat applied to the feed thermostat region (see Fig. 8a) and a second Langevin thermostat applied to the permeate thermostat region to maintain the permeate solution at 290 K. The production run lasts for 4 ns, and the water permeation and salt rejection behaviour during this time frame are recorded for further analysis. Water permeance is measured in terms of number of water molecules entering the permeate region, and salt rejection as a percentage of the number of salt ions entering the permeate region. Water permeability is obtained from scaling methodology detailed in Supplementary Fig. 16.

### Data availability

The data that support the findings of this study are available from the authors upon request.

## Electronic supplementary material

Supplementary Information

Description of Additional Supplementary Files

Supplementary Movie 1

Supplementary Movie 2

Supplementary Movie 3
