# Supplementary material for: Anti-fouling graphene-based membranes for effective water desalination
Source: Nat Commun. 2018 Feb 14;9:683. doi: 10.1038/s41467-018-02871-3 (PMC5813009; doi:10.1038/s41467-018-02871-3)
Supplement: Supplementary file 1 — Supplementary Information [file 41467_2018_2871_MOESM1_ESM.pdf]

Supplementary Information

Anti-fouling graphene-based membranes for effective water desalination by

Dong Han Seo, et al.

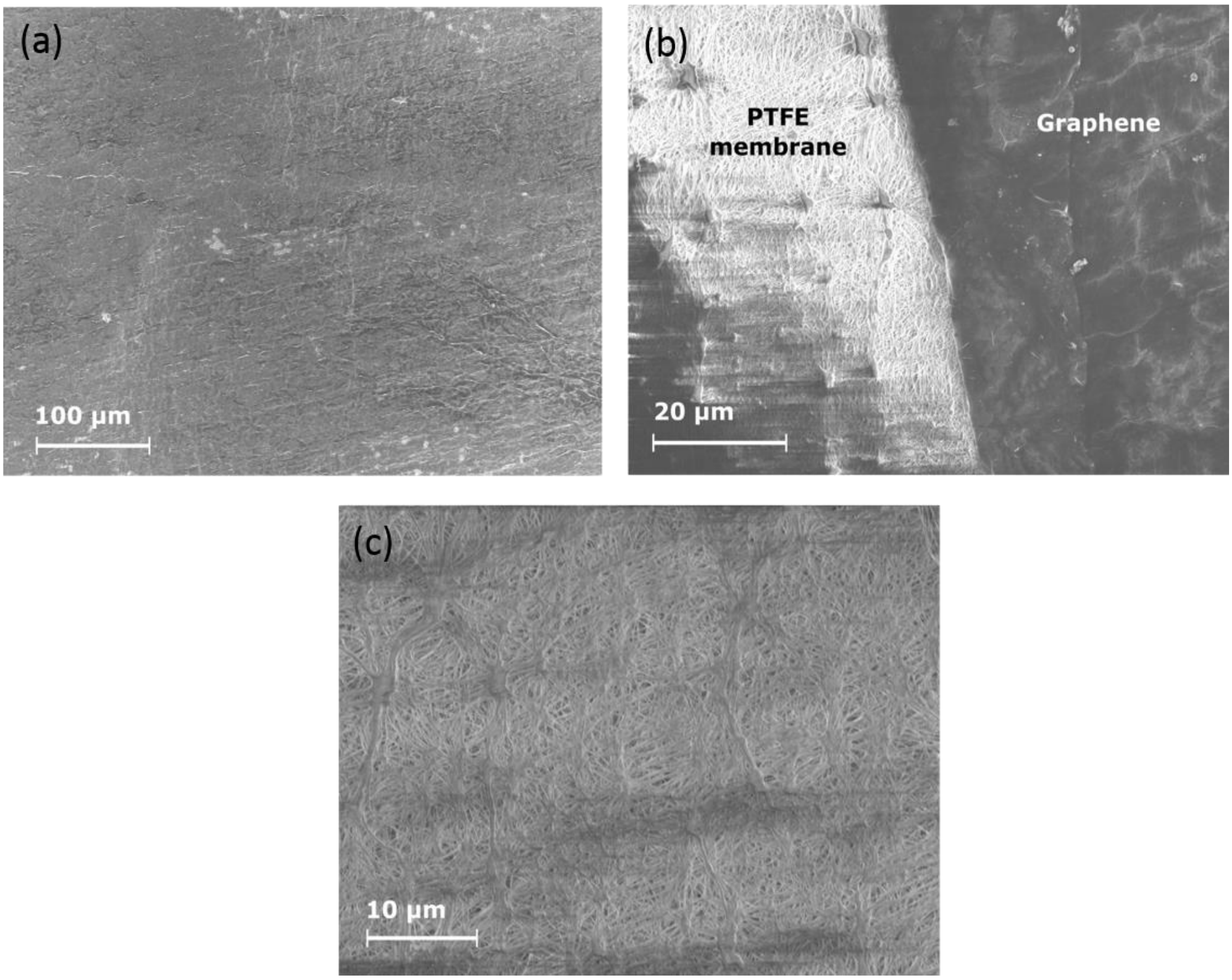

Supplementary Figure 1. Additional SEM images revealing the surface features and morphology of permeable graphene and commercial MD membrane. SEM image revealing large area uniform coverage graphene on top of commercial MD membrane consisting of polytetrafluoroethylene (PTFE) polymer, commercial PTFE based MD membrane/permeable graphene junction and SEM of pristine PTFE based MD membranes. (a) Large-area low-magnification image of permeable graphene film on PTFE membrane. It is evident that many ripple like structures are present. (b) Boundary between graphene and PTFE membrane. (c) Higher-magnification SEM image of PTFE membrane, microporous web-like structure is evident.

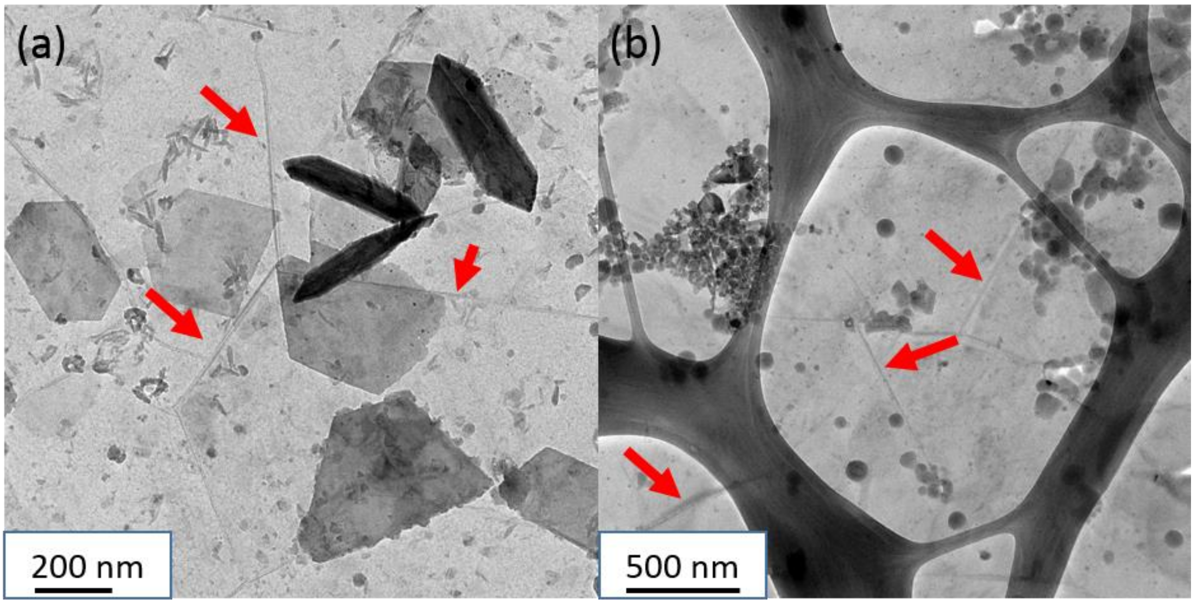

Supplementary Figure 2. Additional TEM images revealing the overlapping of grain boundaries in few-multi layer graphene film used for membrane testing. TEM image revealing large area graphene on Cu TEM grids, (red arrow) pointing to the dark lines on TEM images representing the regions of mismatched overlapping of graphene grain boundaries.

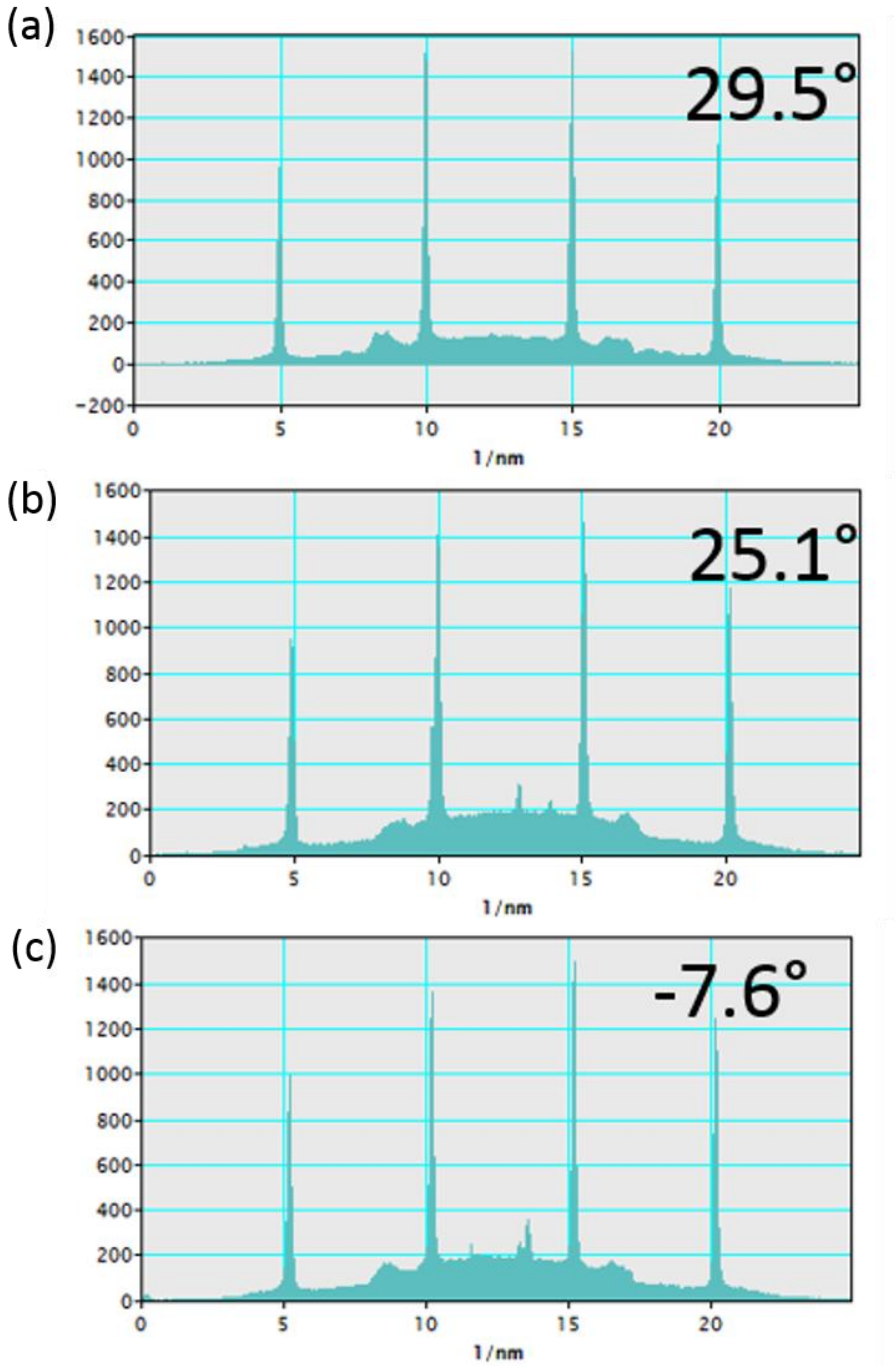

Supplementary Figure 3. Intensity profile from SAED of Fig. 3. (a) Intensity profile from SAED of Fig. 3b, single layer graphene. (b, c) Intensity profile from SAED of Fig. 3d, turbostratic bilayer graphene

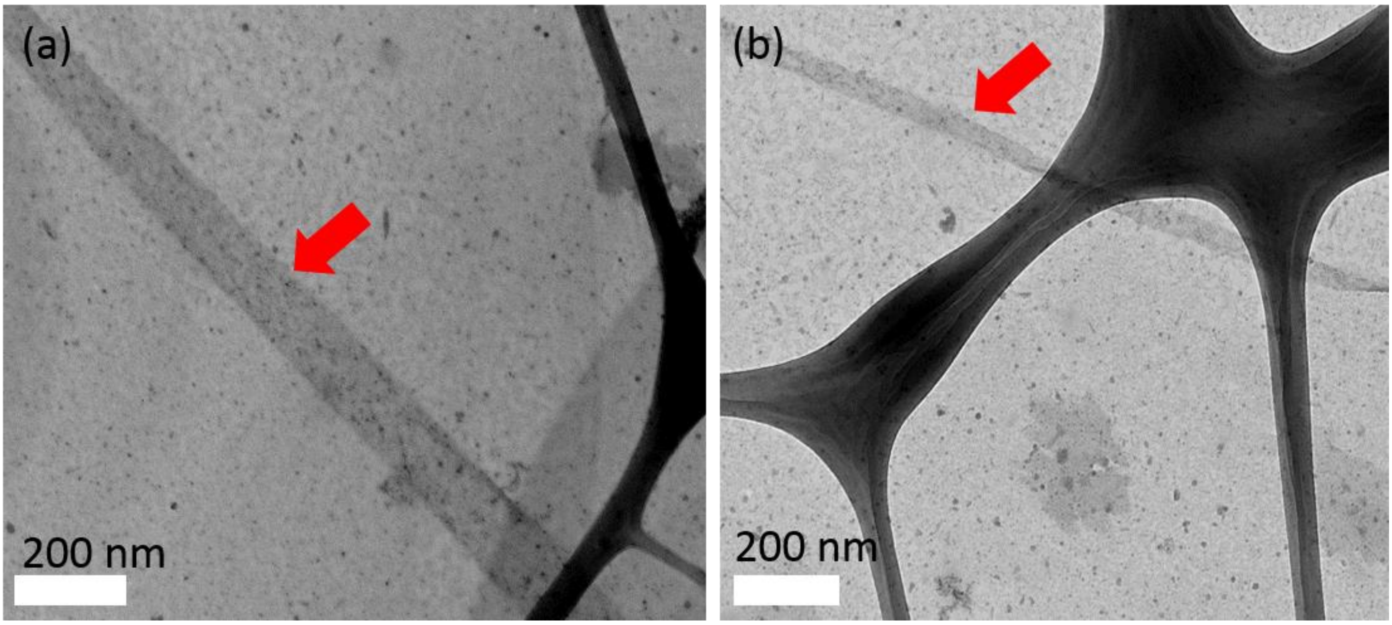

Supplementary Figure 4. TEM of images of predominately single or bilayer graphene with nanochannels. Single to bi-layer graphene with nanochannels were synthesized to clearly demonstrate the existence of the overlapping of graphene domain boundaries. A strip of a darker contrast region is representative of the nanochannels (red arrow).

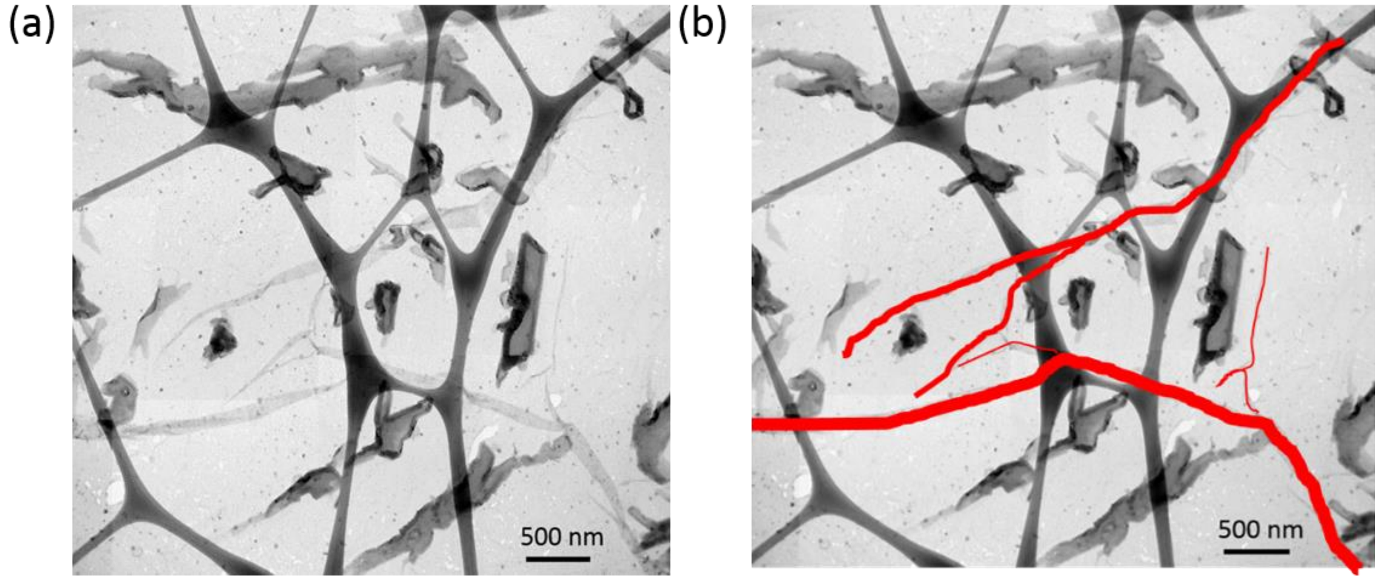

Supplementary Figure 5. A montage of low magnification TEM images of predominately single or bilayer graphene on lacey carbon TEM grid. Regions showing extended lines of darker contrast, and highlighted with red in (b), are either folds of the graphene sheet or overlapping domain boundaries (nanochannels), which can be confirmed through SAED analysis. Multilayers are also visible as regions with darker contrast and defined sharp angled edges.

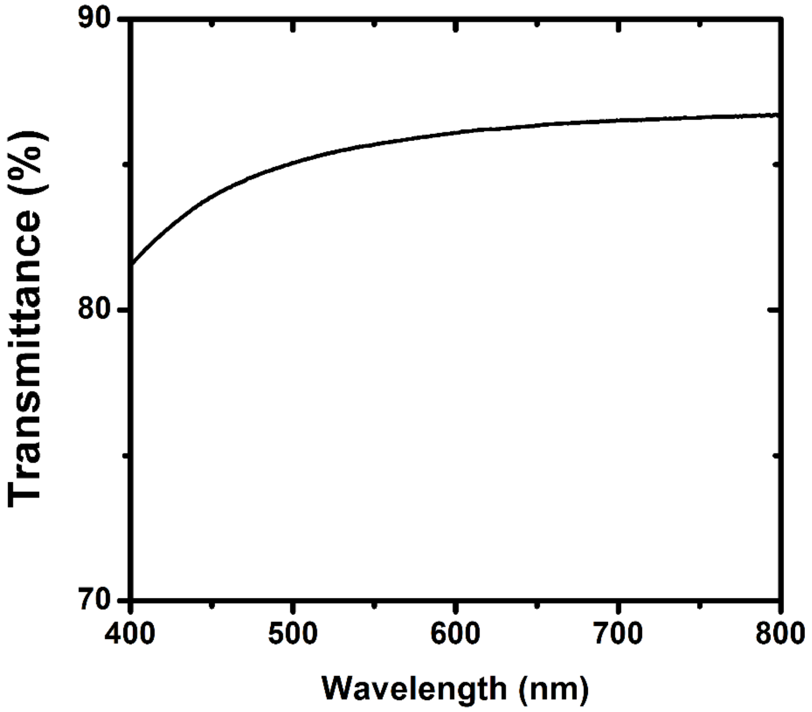

Supplementary Figure 6. Optical transmission spectrum of permeable graphene. Optical transmittance of permeable graphene film taken from glass slide after transfer. Sampling area was 2 cm^2. Transmittance of 85% suggests we have few to multi-layer graphene film.

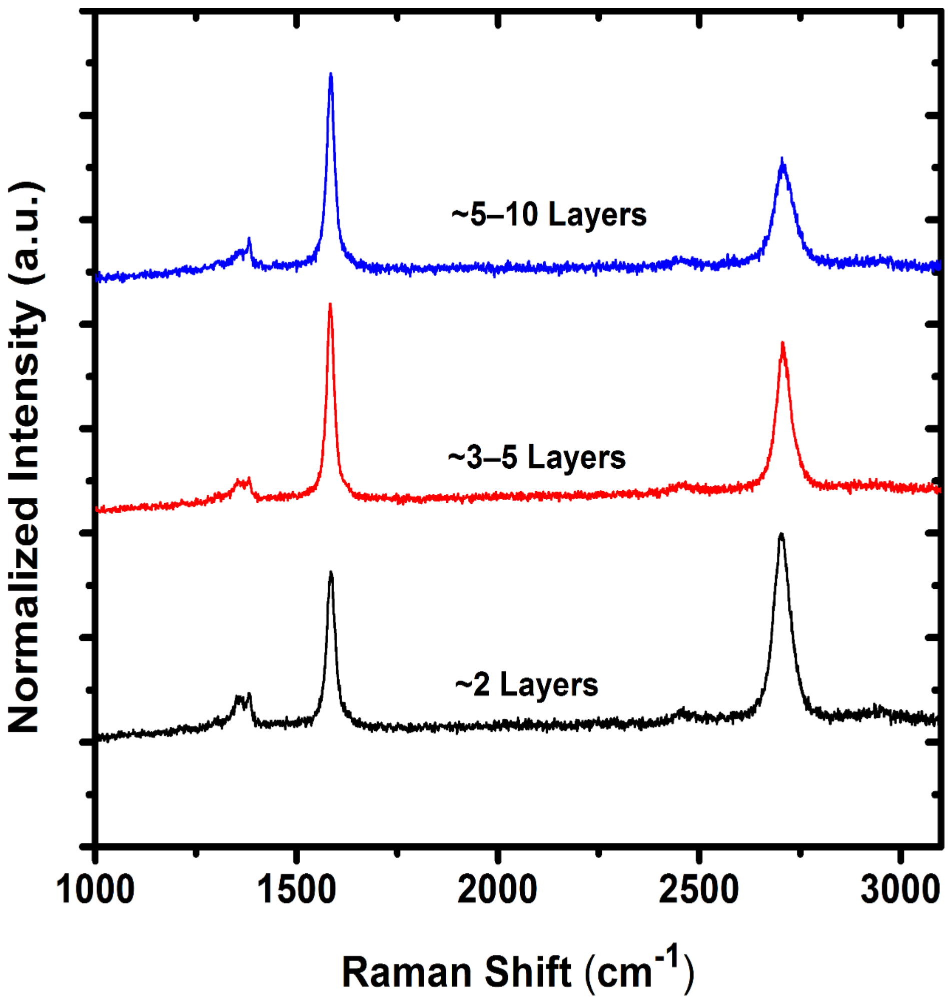

Supplementary Figure 7. Individual Raman spectra taken from selected areas in permeable graphene samples. Raman spectra suggest presence of multi-layer graphene with variation in number of layers in graphene.

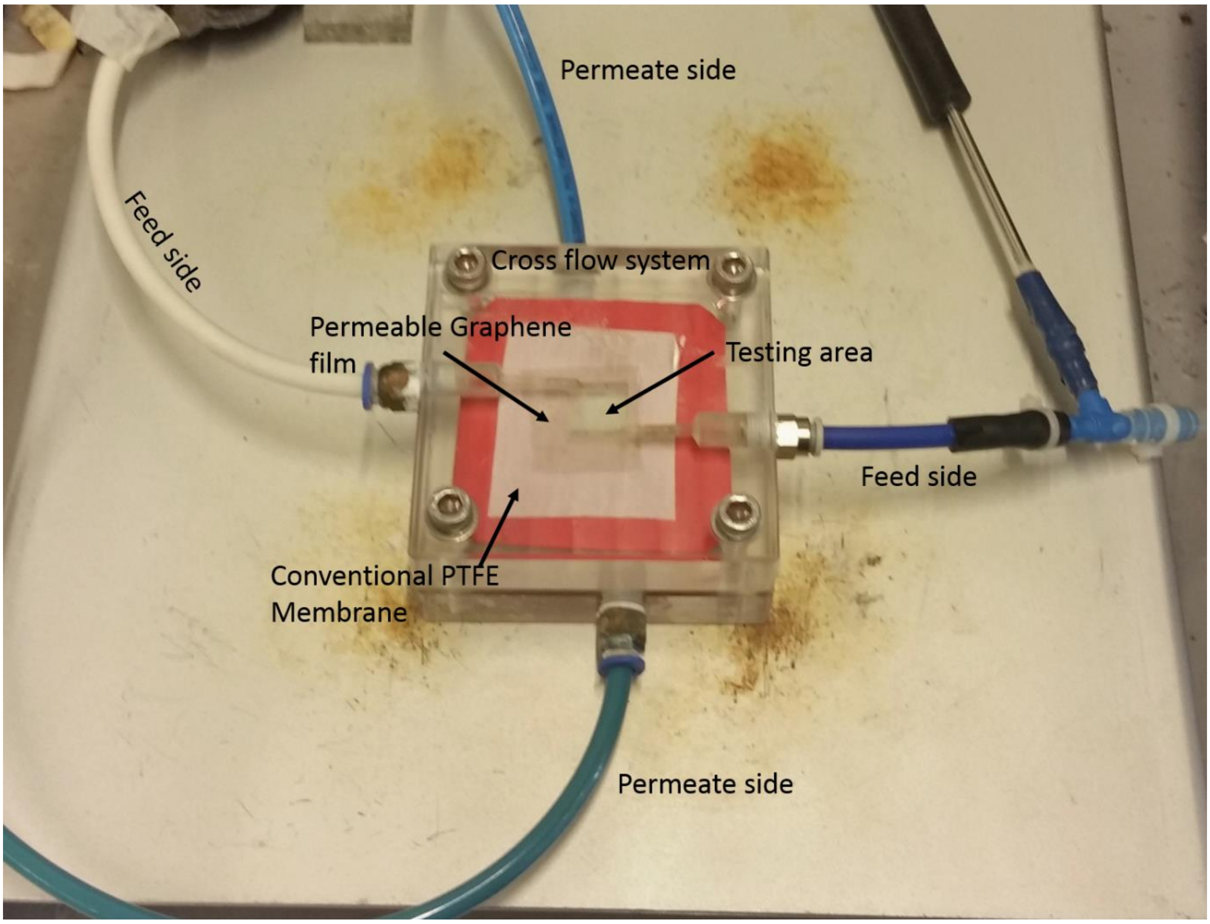

Supplementary Figure 8. Testing set up for the water desalination and purification. Testing was carried out in a continuous cross flow system where permeable graphene film was placed in between the feed and the permeate side.

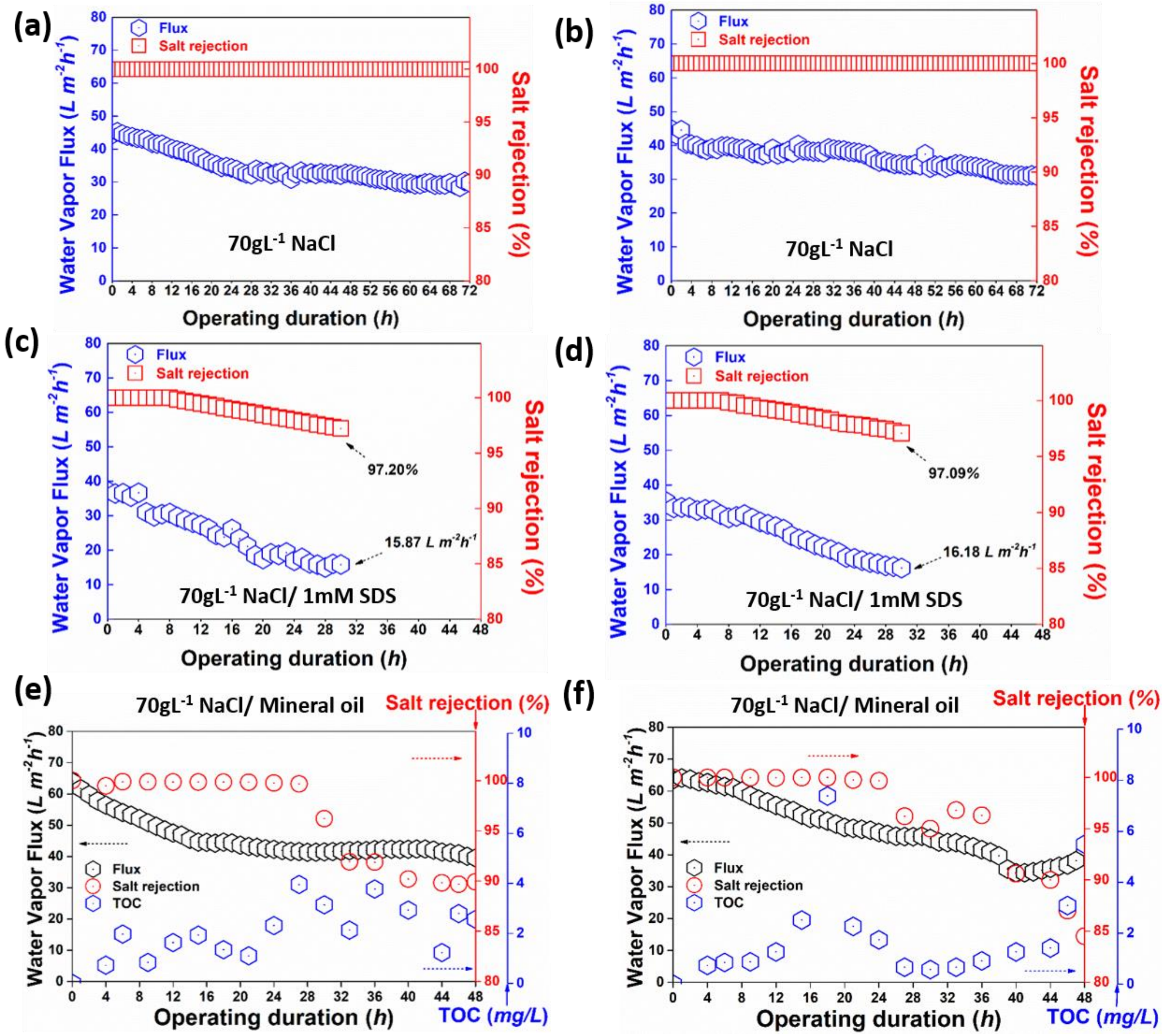

Supplementary Figure 9. Repeated MD experiments of pristine PTFE based MD membrane with saline water, SDS/Saline water mixtures and mineral oil/Saline water mixtures. All the fouling experiments were repeated twice to demonstrate the reproducibility of pristine PTFE based membrane performance. (a,b) demonstrate the repeated experiments with saline water (70 gL^{-1} of NaCl), (c,d) demonstrate the repeated experiments with SDS/saline water mixtures (1mM SDS/ 70 gL^{-1} of NaCl). The result shows rapid degradation of membrane performance is observed. Similarly, (e, f) demonstrate the repeated experiments with mineral oil/Saline water mixtures (1 gL^{-1} mineral oil with 70 gL^{-1} of NaCl and 1 mM $NaHCO_3$). The result shows, significant reduction in water vapor flux was observed, with degradation in salt rejection to 85~90% over 48 hours with increasing in TOC level demonstrating the passage of oil through the membrane.

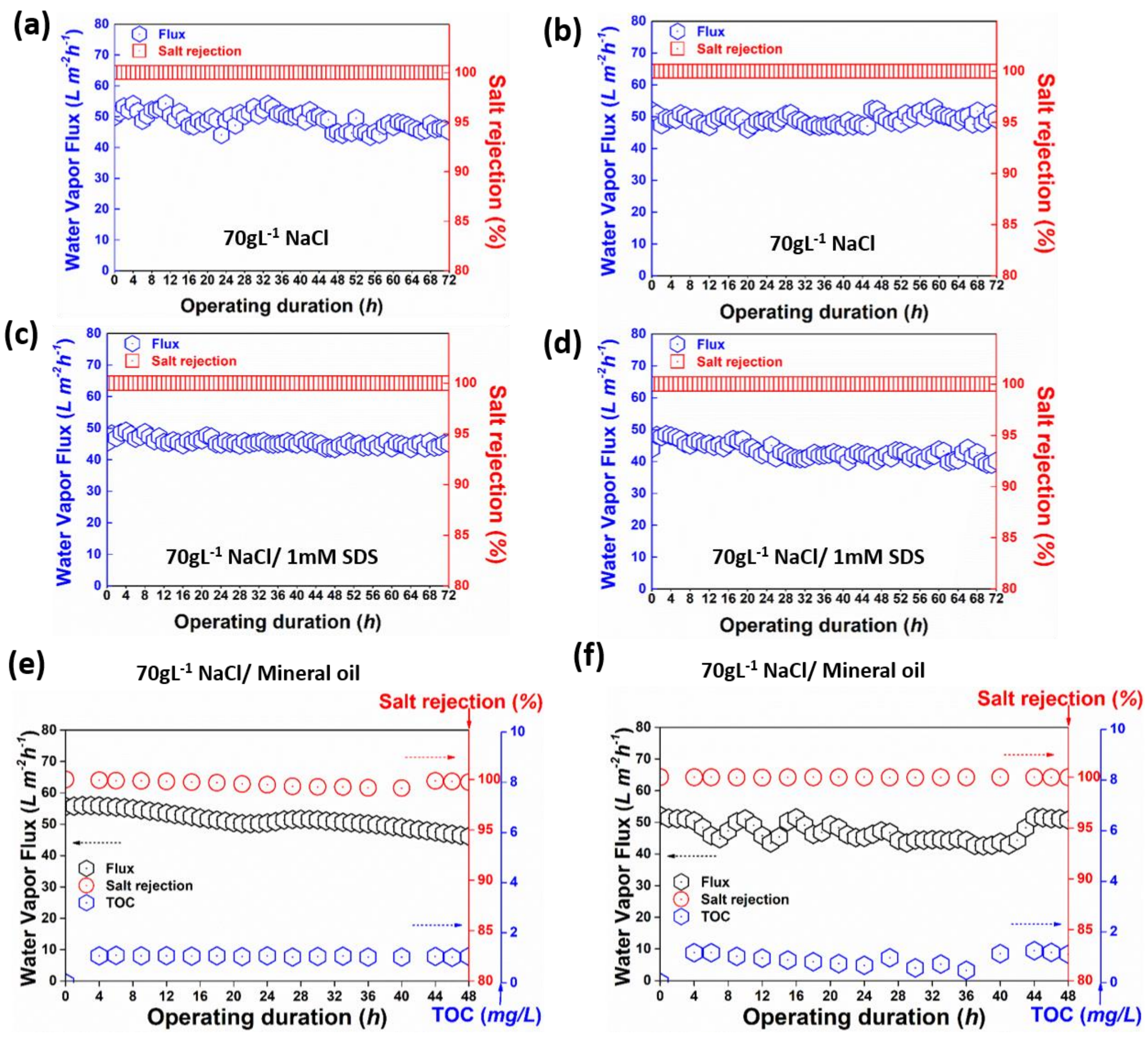

Supplementary Figure 10. Repeated MD experiments of permeable graphene based membrane with saline water, SDS/Saline water mixtures and mineral oil/Saline water mixtures. All the fouling experiments were repeated twice to demonstrate the reproducibility of permeable graphene based membrane performance. (a,b) demonstrate the repeated experiments with saline water (70 gL^{-1} of NaCl), (c,d) demonstrate the repeated experiments with SDS/saline water mixtures (1mM SDS/ 70 gL^{-1} of NaCl). The result shows stable water flux with >99.9% salt rejection is achieved for 72 hours of MD operation. Similarly, (e, f) demonstrate the repeated experiments with mineral oil/Saline water mixtures (1 gL^{-1} mineral oil with 70 gL^{-1} of NaCl and 1 mM $NaHCO_3$). In this case, we also monitored TOC of the permeate water to show the oil rejection over 48 hours of MD operation. The result shows, slight reduction in water vapor flux was observed, with salt rejection of >99.9% along with stable oil rejection over 48 hours.

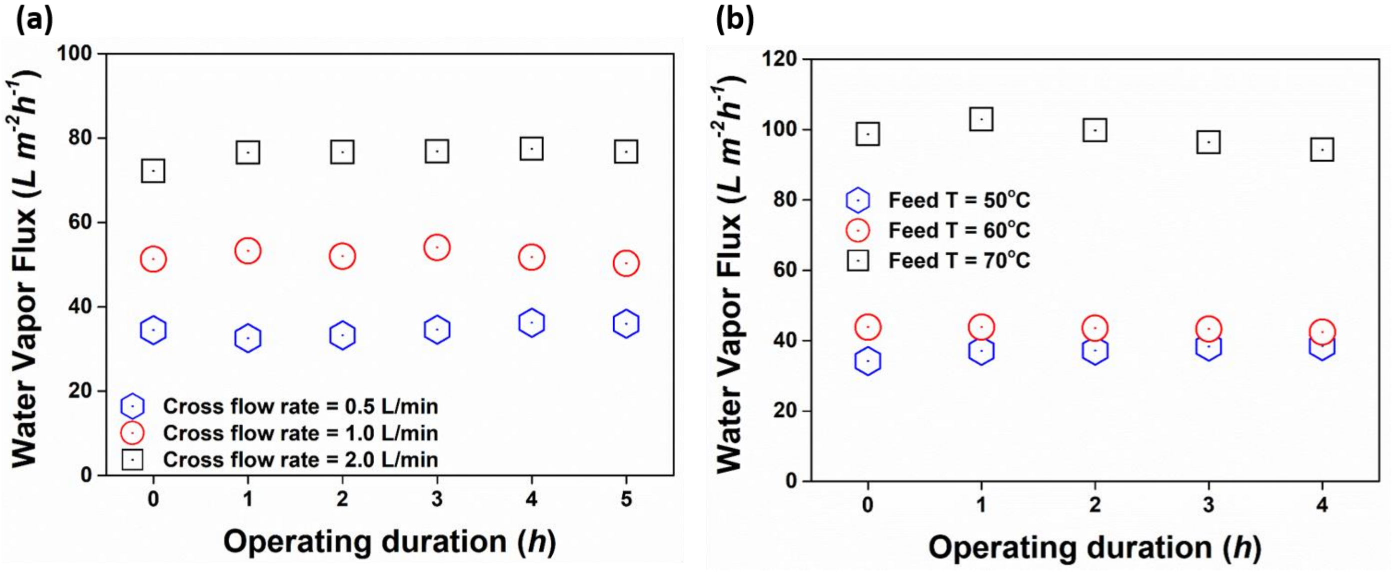

Supplementary Figure 11. Membrane performance of permeable graphene at different cross flow and with different feed temperatures. To investigate the factors affecting the water vapor transport in permeable graphene based membrane, we varied the different process parameters such as (a) cross flow rate of supplied water and (b) feed water temperatures to see the changes in water vapor permeation. The results show that water vapor flux increased as the cross flow rate of the water stream was increased. Similarly, as the temperature of the feedwater was increased, water vapor flux was also increased. In all cases, stable water vapor flux was maintained at the permeate side for the permeable graphene based membrane throughout the duration of MD operation. All the tests were done with saline solution (70 gL^{-1} of NaCl).

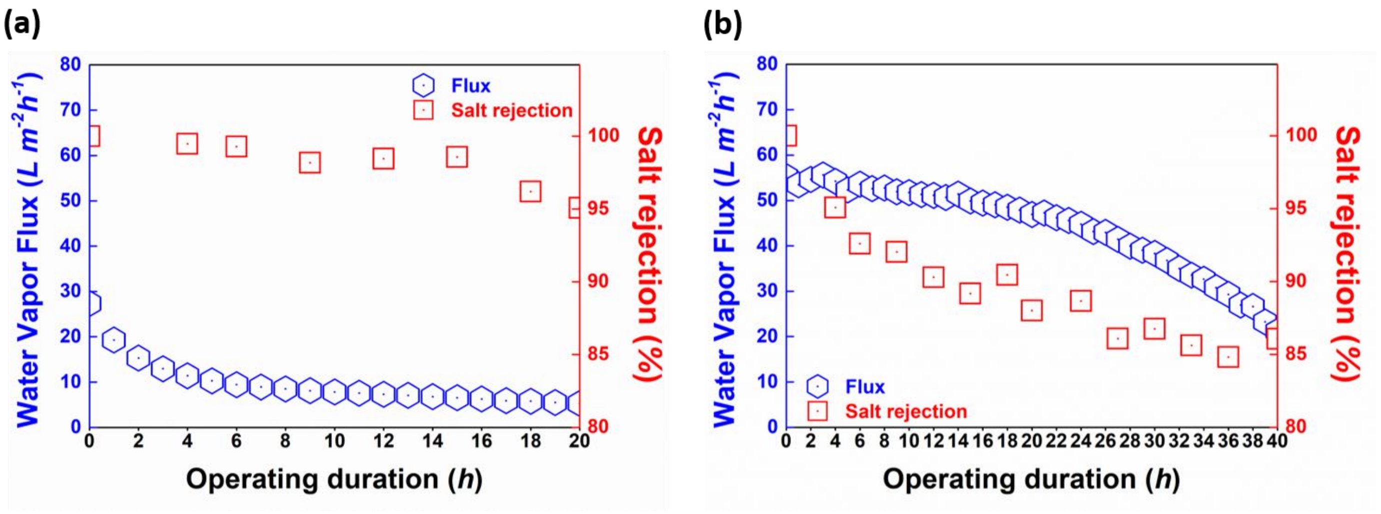

Supplementary Figure 12. Commercial PVDF based MD membrane test with mineral oil/saline water mixture and SDS/saline water mixtures. Another widely used MD membrane is PVDF based MD membrane (Durapore). Commercial PVDF based membrane test under (a) mineral oil/saline water mixture and (b) SDS/saline water mixtures was performed to demonstrate the fouling problem against low surface tension liquids are not just restricted to PTFE based MD membrane but it is a general problem for MD membranes. The results shows that significant flux reduction is observed for both cases of (a) mineral oil/saline water mixture and (b) SDS/saline water mixtures along with decrease in salt rejection, showing the membrane failure within a short MD operation period.

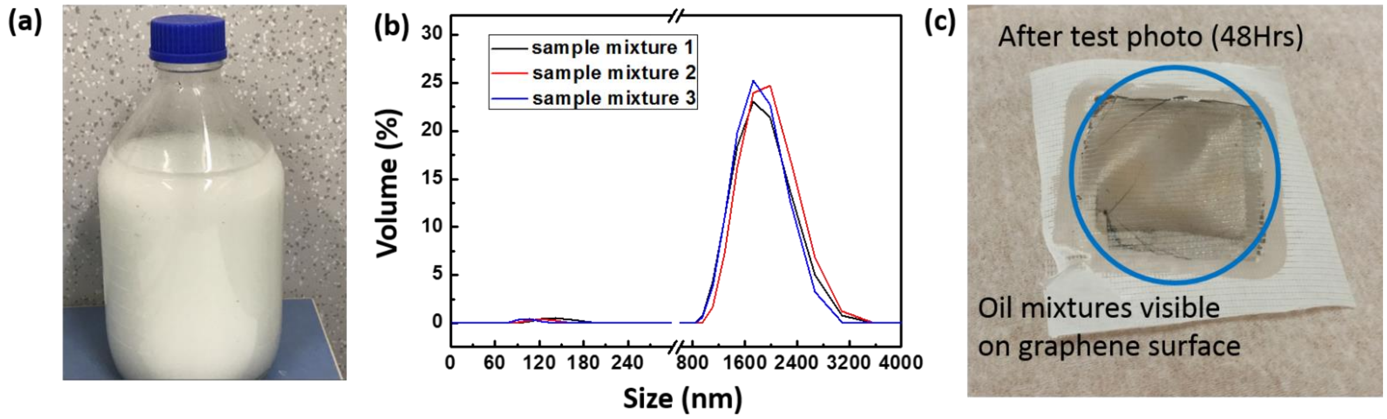

Supplementary Figure 13. Mineral Oil/Saline water mixture used for experiments with particle size distribution and after test photo of permeable graphene with mineral oil/saline water mixture. (a) shows the photo graph of the mineral oil/saline water mixture used in the experiments, as one can see stable oil emulsion has been formed. (b) shows the oil size distribution curve showing oil contents were mostly 1 to3 µm in sizes along with minority content with sizes from 78 nm to 180 nm. (c) is after-test photograph of the permeable graphene. Over 48 hours of filtration tests, significant oil contents are visible on the surface of the graphene film.

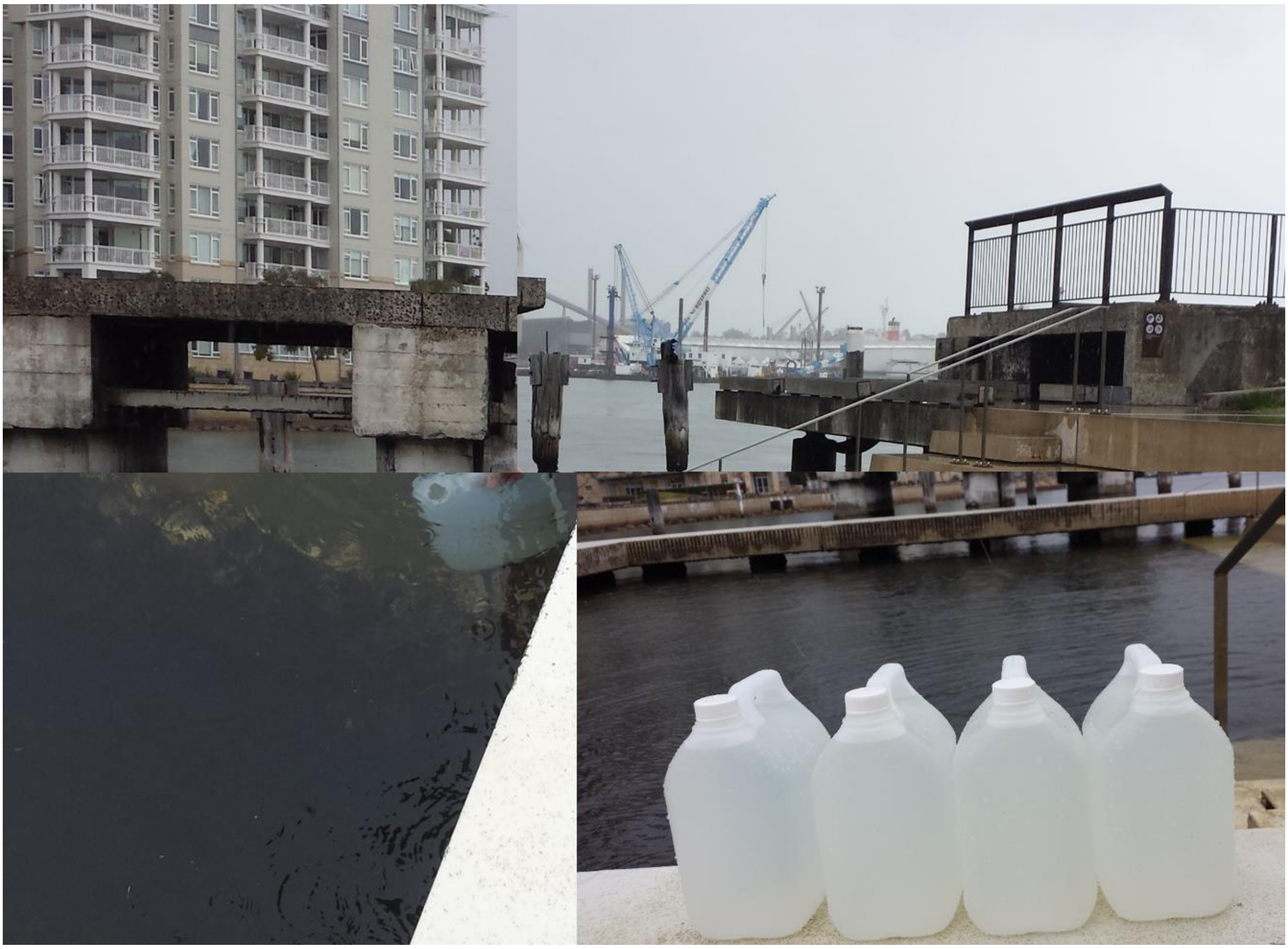

Supplementary Figure 14. Extraction site for seawater samples from Sydney Harbour. Both household and industry sites are evident near the seawater extraction site. Water was used directly from the source without pre-cleaning or filtration to demonstrate the capability of permeable graphene based membrane in real desalination situation.

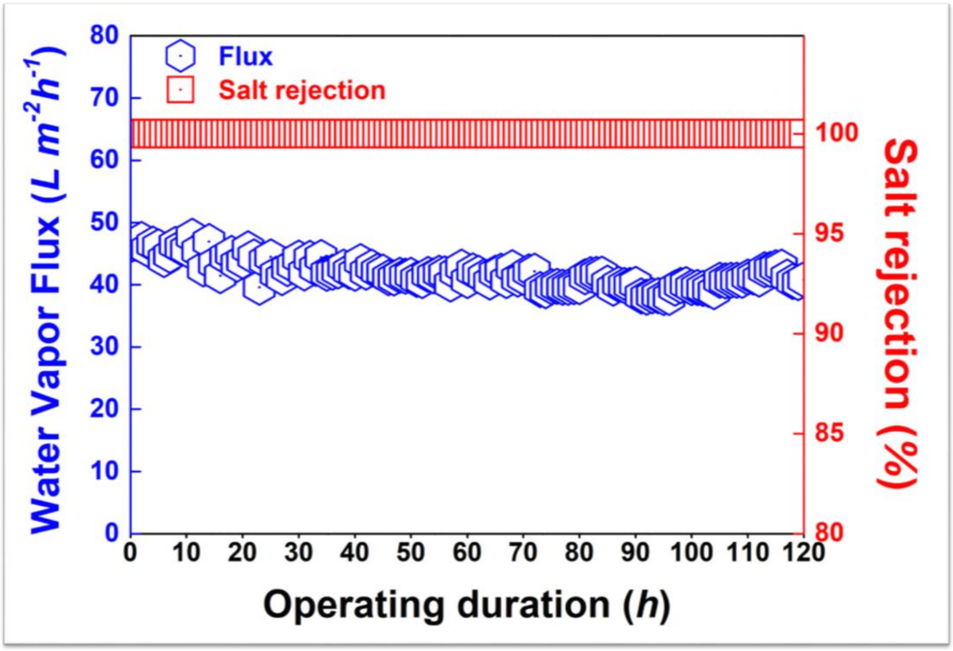

Supplementary Figure 15. Long term membrane performance of permeable graphene with sea water collected from Sydney Harbour for 120 hours. Long term (120 hours, 5 days) membrane performance test was performed with sea water collected from Sydney Harbour to demonstrate the practical applicability and long term stability of permeable graphene based membrane. The results show that through permeable graphene, we obtained stable water flux as well as stable salt rejection of > 99.9% over 120 hours of MD operation revealing permeable graphene's excellent capability as anti-fouling, long term stable membrane material.

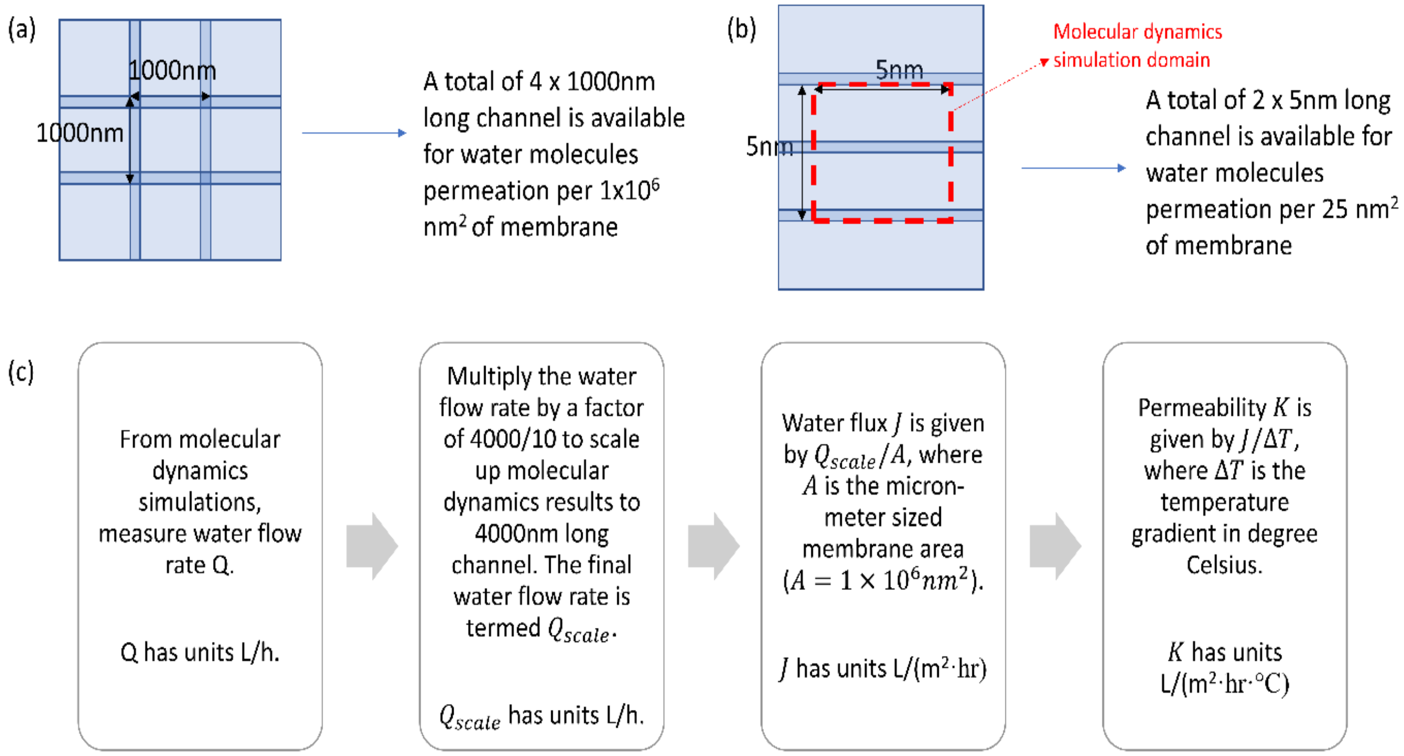

Supplementary Figure 16. Scaling methodology to scale permeability results from molecular dynamics simulations to micro-meter sized permeable graphene-based membrane. (a) Simplified schematic diagram representing the deduced structure of a micro-meter sized permeable graphene-based membrane from experimental characterizations. Darker areas indicate overlapping regions. (b) Schematic diagram illustrating the membrane constructed in the molecular dynamics simulations. (c) Process chart depicting the scaling methodology employed from the water flow rate Q measured during molecular dynamics simulation, to the estimated permeability K of a micro-meter sized permeable graphene-based membrane. This scaling methodology is used to construct the graph shown in Fig. 8d.

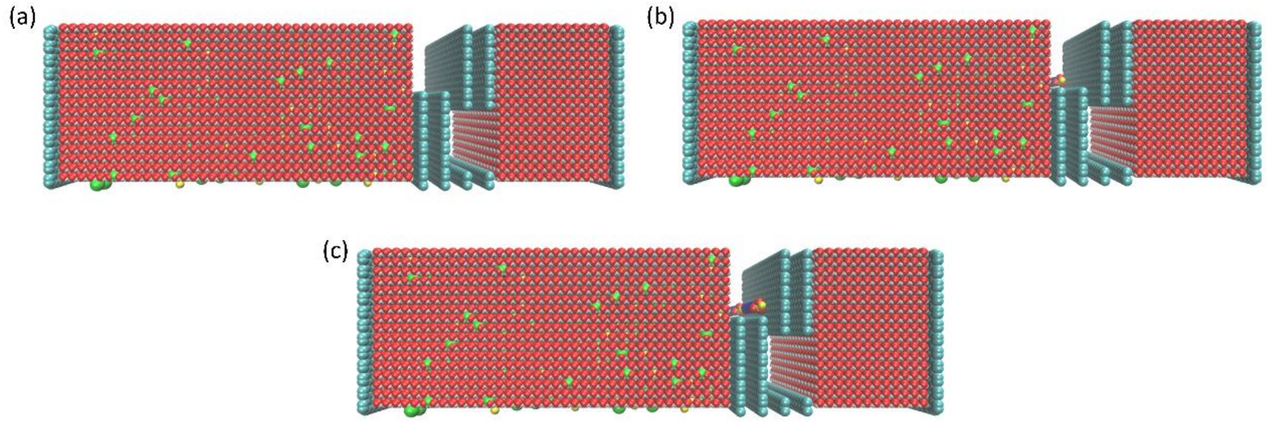

Supplementary Figure 17. Initial configurations used to start the molecular dynamics simulations. Each molecular dynamic simulation starts with a configuration where the salt ions are randomly distributed in the feed region, while the SDS molecules are placed right at the channel entrance. The configurations shown are for feed solution containing (a) 2M NaCl, 0M SDS (b) 2M NaCl, 5mM SDS (c) 2M NaCl, 10mM SDS. This initial positioning of the SDS molecules helps save on the computational time required for the SDS to move to the channel entrance, and it represents the most extreme scenario in which the SDS molecules are most likely to affect the permeability performance of the membrane.

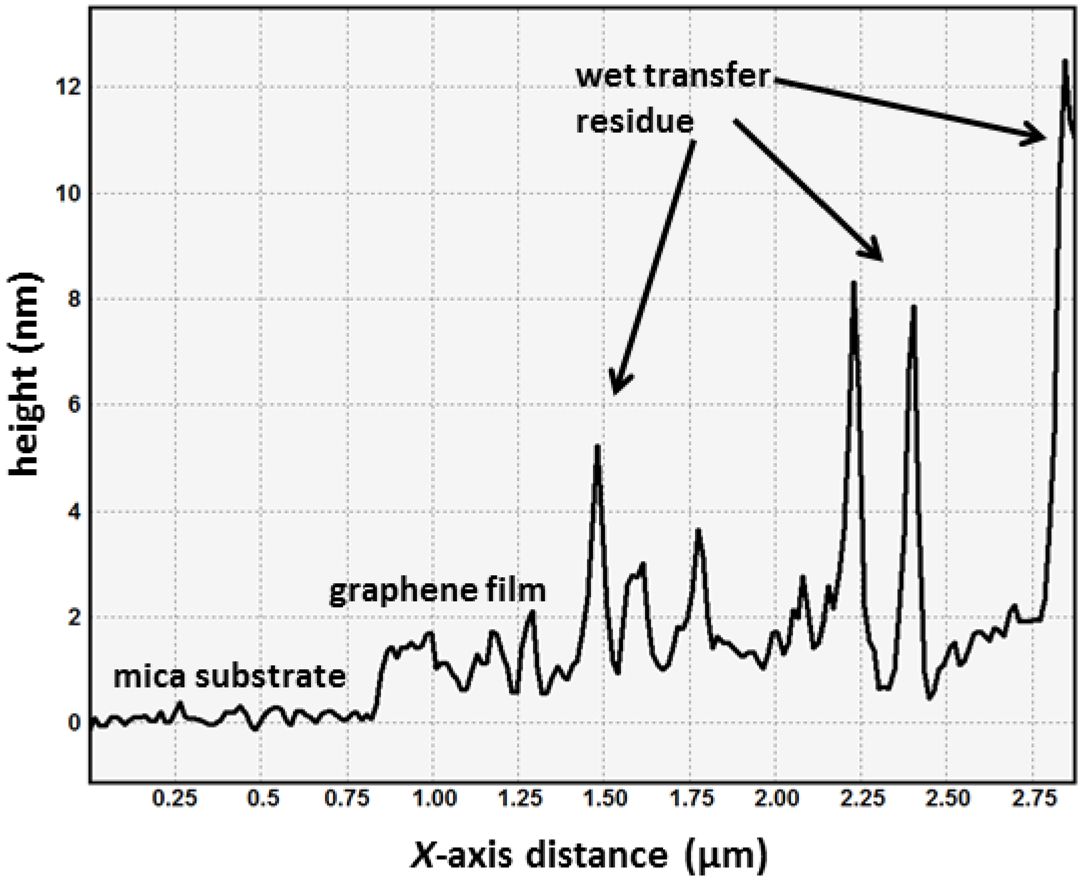

Supplementary Figure 18. AFM topography measurement of a permeable graphene film. The cross section profile of the graphene film on the mica substrate was extracted from Fig 3a. AFM topography measurement indicates that the permeable graphene film surface is rough, which is reflected by the variation in the height of the graphene film ranging from 0.7 nm to 3.7 nm. Wet transfer residue were several nm high. Rough surface of permeable graphene film creates favourable morphology for water vapor permeation.

Feed temperature 90ºC/Permeate temperature 20ºC

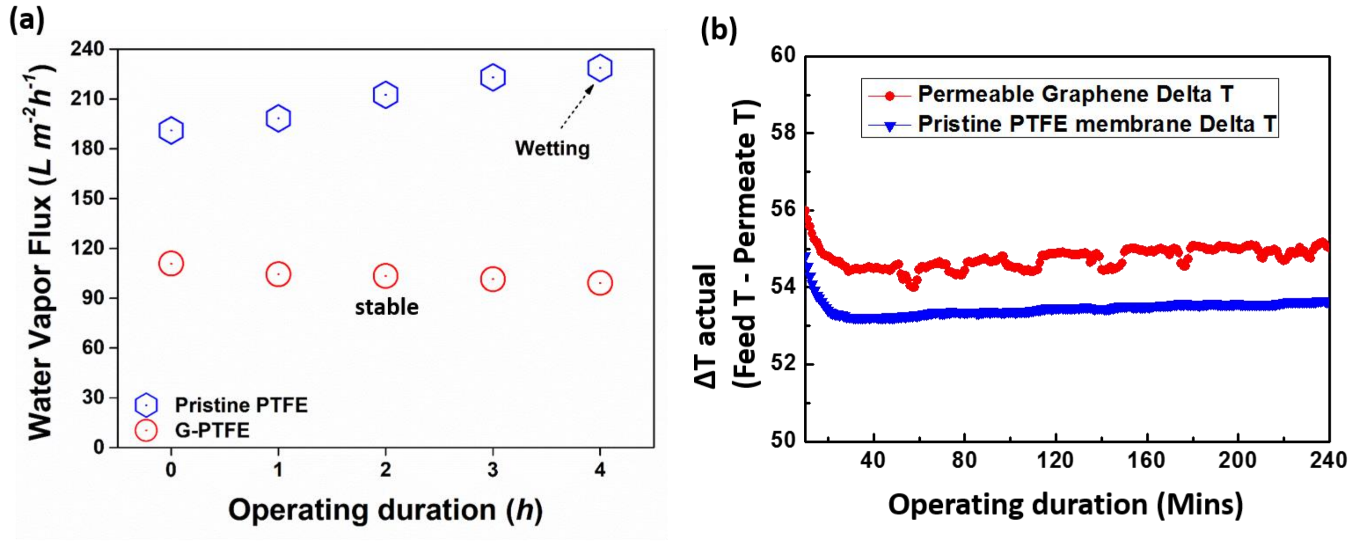

Supplementary Figure 19. Additional feature of permeable graphene based membrane. To explore the additional advantages of incorporating permeable graphene film in the MD membrane, we carried out a MD experiment with high (90ºC) temperature of feed water. Then the permeated vapor flux and the temperature of the feed at the surface of the membrane and permeated vapor temperature were recorded over 4 hours of MD operation. All the tests were done with saline solution (70 gL^{-1} of NaCl). Then the difference in temperature of the actual feed water and permeated vapor flux were calculated over MD operation time. Firstly, (a) water vapor flux curve shows that wetting behaviour for pristine PTFE membrane, where we observed a sharp increase in water vapor flux over 4 hours, the membrane is failing to maintain stable performance at high feed temperature. However, when permeable graphene film was incorporated, high and stable water vapor flux was maintained for the duration of MD operation, demonstrating superior membrane stability of permeable graphene based membrane under high temperature gradient, which can potentially widen the stable operation temperature window of the MD process. (b) shows the actual temperature difference recorded for the pristine PTFE membrane and permeable graphene based membrane. The results show that permeable graphene was able to maintain a higher temperature gradient compared to pristine PTFE membrane throughout the duration of MD operation, demonstrating potential thermal benefits of using graphene film in MD process.

Pristine PTFE membrane

(a)	Tensile Stress at Maximum Load/Yield (MPa)	Tensile Stress at Break (MPa)	Extension at Maximum Load (mm)
1	0.0173	0.0173	0.3802
2	4.2587	1.7707	6.1550
3	0.0173	0.0173	0.0000
4	0.0173	0.0173	0.0722
5	4.9027	3.2920	5.9447
6	2.9880	0.5187	5.0467
7	0.0173	0.0173	0.0225
8	0.0173	0.0173	0.0049
9	3.4893	0.3933	5.3870
10	2.5053	0.0173	5.9303
11	3.2747	0.8227	3.6886
12	0.0173	0.0173	1.4635

Permeable graphene/PTFE membrane

(b)	Tensile Stress at Maximum Load/Yield (MPa)	Tensile Stress at Break (MPa)	Extension at Maximum Load (mm)
1	6.1733	1.9320	4.1050
2	2.6307	0.0173	4.0473
3	4.3667	2.7733	6.1297
4	3.8120	0.1600	4.8313
5	0.0173	0.0173	0.3795
6	0.0173	0.0173	0.2305
7	0.0173	0.0173	0.4552
8	5.0827	3.4720	4.9973
9	4.2773	4.0080	5.3467
10	1.0013	1.0013	1.6386
11	2.0213	0.1787	4.5721
12	2.1293	1.7173	4.9556

Supplementary Figure 20. Mechanical strength measurement of permeable graphene/PTFE membrane and pristine PTFE membrane. To investigate the changes in mechanical strength of the membrane after the permeable graphene incorporation, mechanical strength tests were performed for the (a) pristine PTFE membrane and after the (b) permeable graphene incorporation. The results show the marginal improvement in the mechanical strength of the membrane when permeable graphene film is incorporated. Improvement was marginal due to thin nature of the permeable graphene film (few nm thick) compared to the bulk (120 µm thick) PTFE membrane.

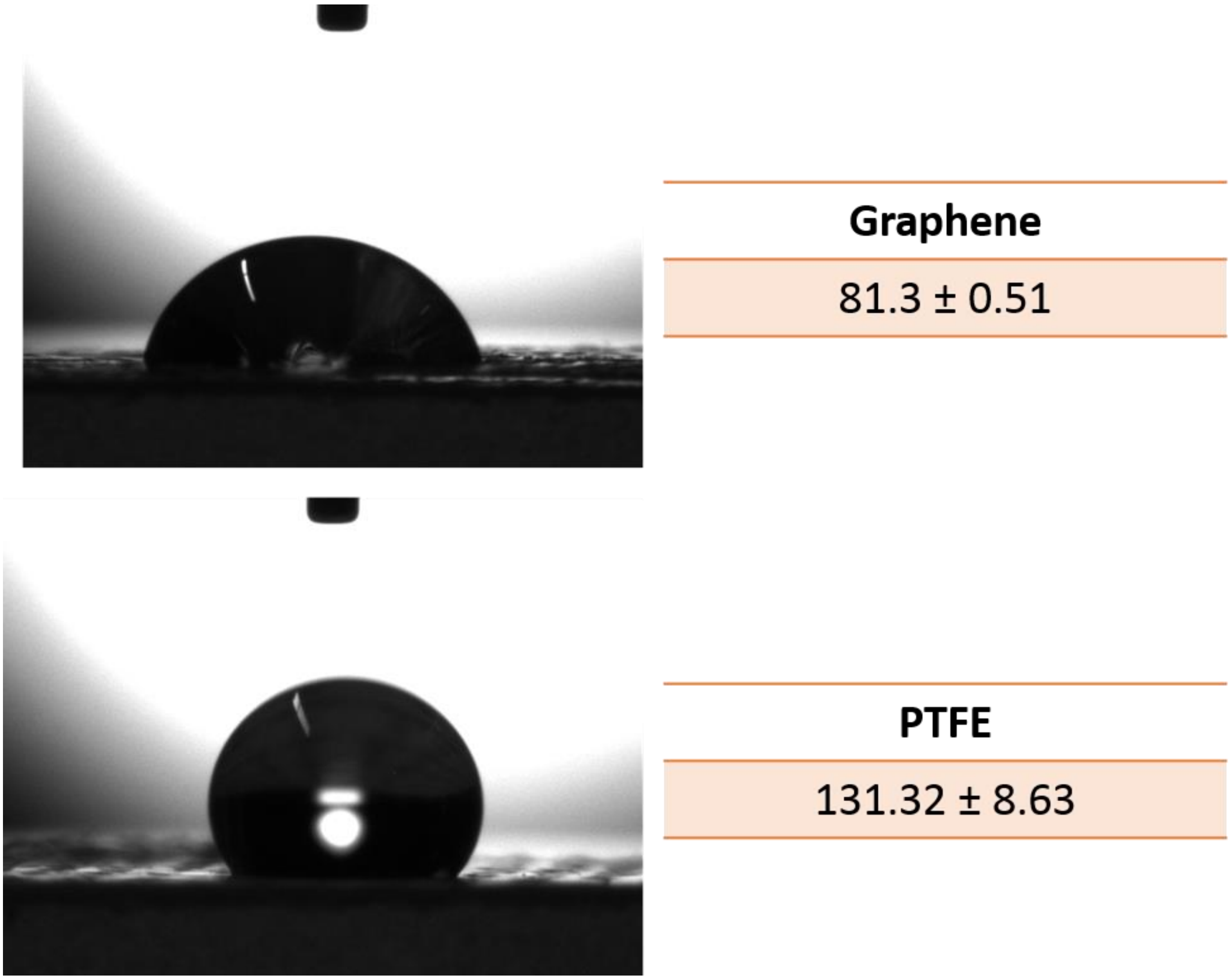

Supplementary Figure 21. Contact angle measurements of permeable graphene film/PTFE membrane and commercial PTFE membrane. Top: Graphene/PTFE membrane. CA 81.3 +/- 0.51 deg. Bottom: PTFE membrane only. CA 131.32 +/- 8.63 deg. Permeable graphene film is shown to be more hydrophilic than PTFE membrane.

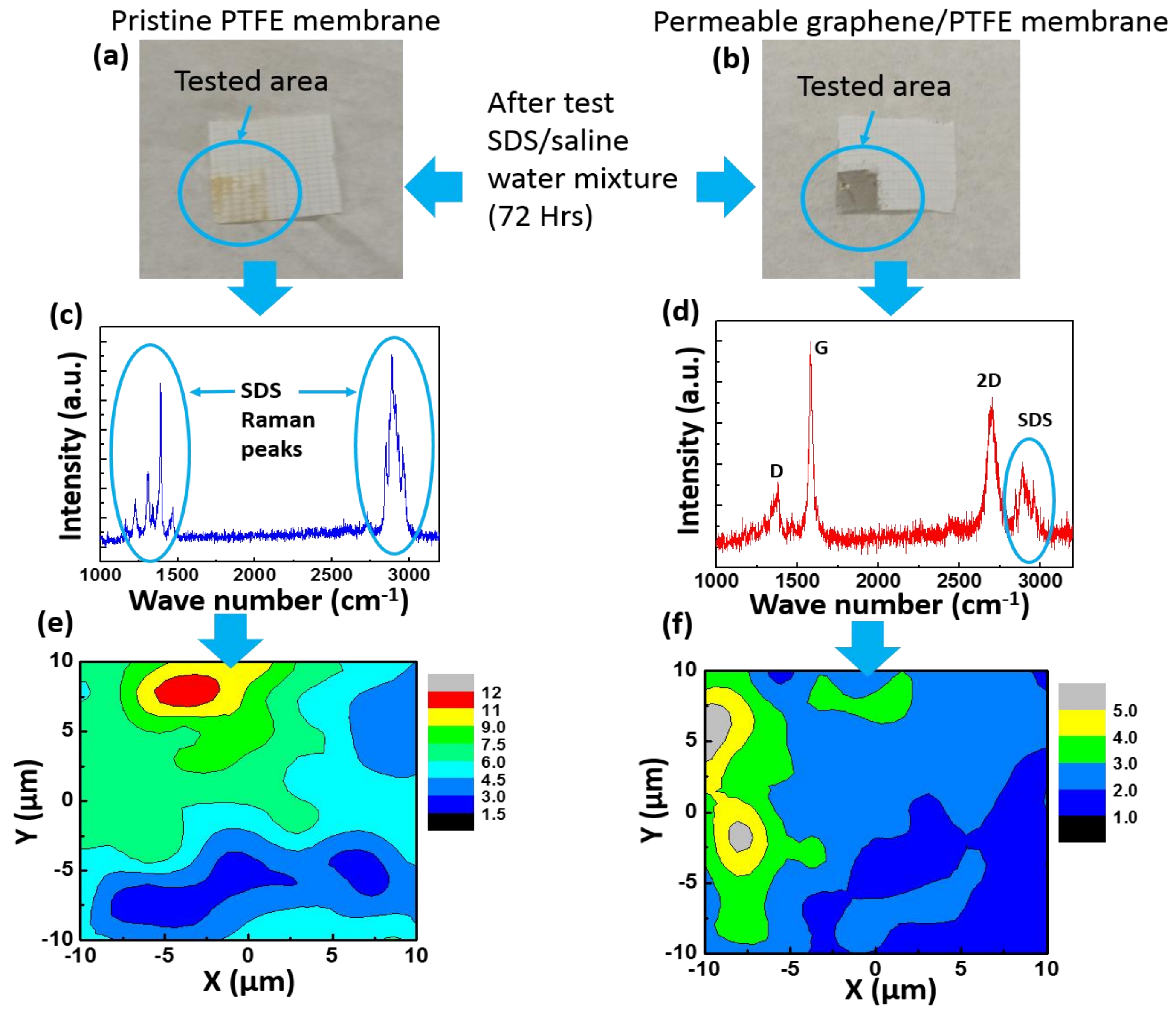

Supplementary Figure 22. Raman analysis of the after test samples under SDS/saline water mixtures. To qualitatively visualise different adsorption behaviour of the SDS to pristine PTFE membrane and permeable graphene surface, Raman analysis was performed on after test (72 hours) samples which were tested under SDS/saline water mixtures. (a, b) shows the part of the after test samples of (a) pristine PTFE membrane and (b) permeable graphene/PTFE membrane. (c, d) shows the individual Raman spectrum of after test (c) pristine PTFE membrane and (d) permeable graphene/PTFE membrane. Then to qualitatively verify the differences in adsorption behaviour, Raman areal mappings of SDS peak intensities were carried out on after test (e) pristine PTFE membrane and (f) permeable graphene/PTFE membrane samples. The results show that significantly higher SDS peak intensities were observed for pristine PTFE membrane compared to the permeable graphene/PTFE membrane case. These findings suggest a significantly higher SDS adsorption on pristine PTFE membrane and reveal different adsorption interactions between SDS molecules with PTFE membrane surface and the permeable graphene surface.

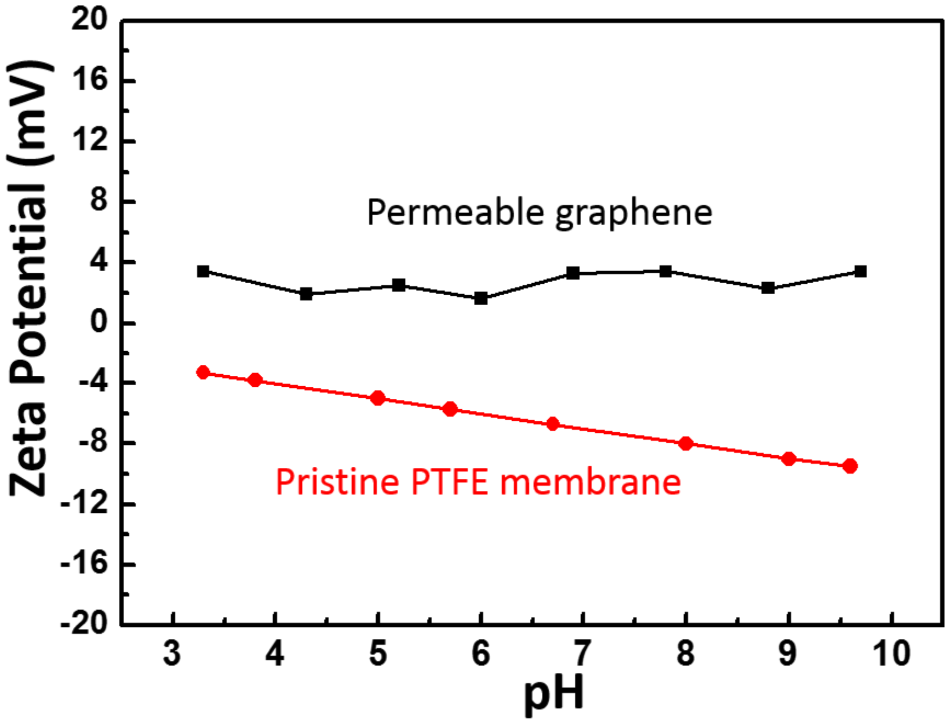

Supplementary Figure 23. Zeta potential measurement of permeable graphene and pristine PTFE membrane. Zeta potential measurements show that our graphene films exhibit almost negligible charge (charge neutral) under varying pH conditions shown by near flat line around 2-4 mV with varying pH conditions. A pristine PTFE membrane shows negative surface charge under varying pH condition.

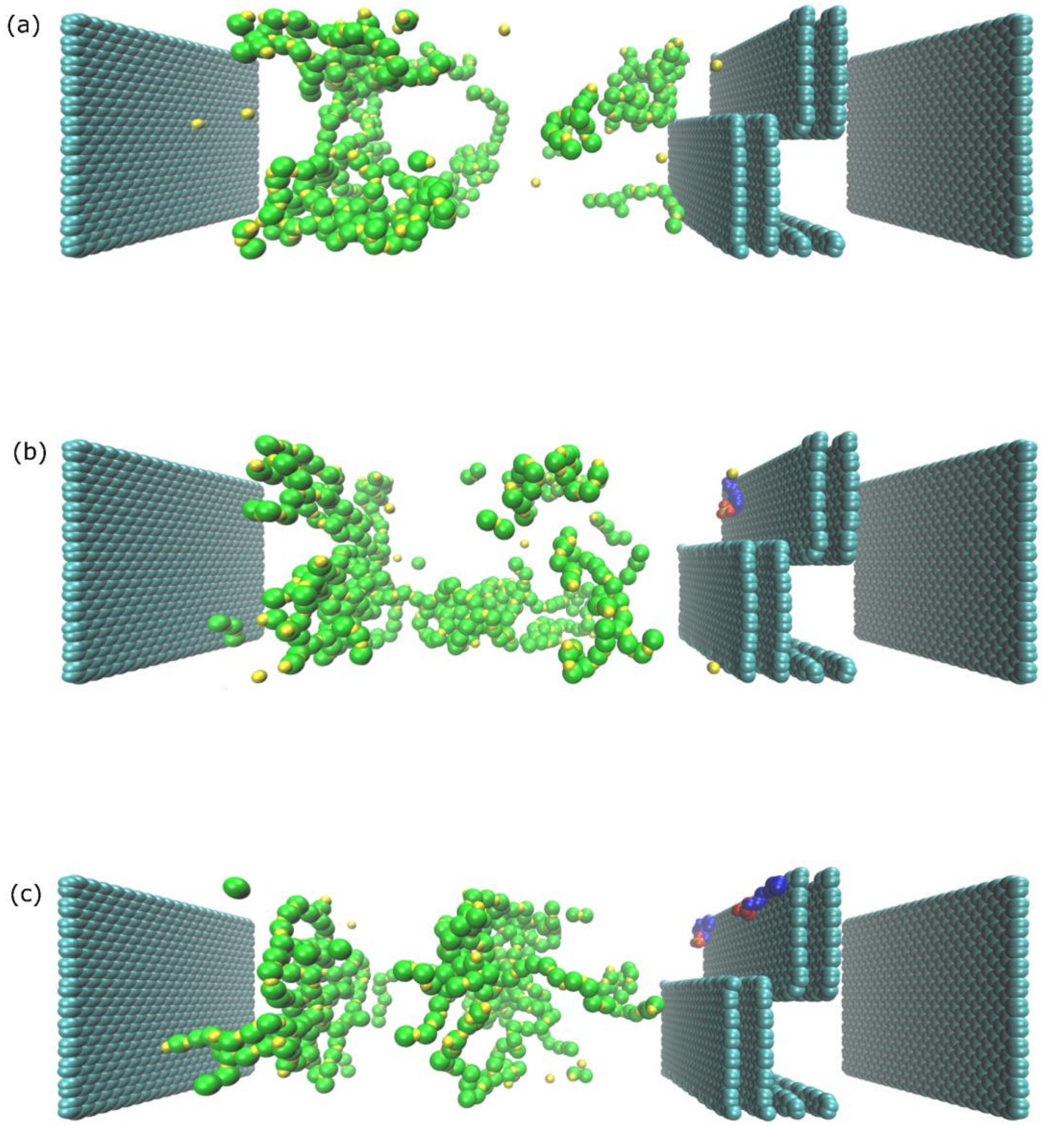

Supplementary Figure 24. Distributions of the Na, Cl and SDS ions at the end of 4ns of Molecular Dynamics simulations. Snapshots taken at the end of the 4ns production run for the molecular dynamics simulation study with feed configuration (a) 2M NaCl, 0M SDS (b) 2M NaCl, 5mM SDS (c) 2M NaCl, 10mM SDS. The water molecules are not shown in these snapshots, C is in cyan, Na is in yellow, Cl in green, O in red, S in pink, and the CH_2 or CH_3 groups are in blue.

Supplementary Table 1. Cost analysis of integrating 1 cm^2 of permeable graphene onto PTFE membrane (US$).

Cost break down	Permeable Graphene
**Precursor material**	Soybean oil used 0.17ml for 9 cm^2 sample<br>$0.00025 (per run)
**Growth substrate**	Ni (25 µm, 99%, Alfa Aesar, 9 cm^2) $0.35
**Electricity for furnace heating**	29 mins in total<br>$0.33 (per run)
**Operation of vacuum pump**	$0.051 for 29 mins<br>(per run)
**Chemicals for transfer**	PMMA (Sigma Aldrich), $FeCl_3$ (Sigma Aldrich), Acetone (Septone)<br>$0.1, $0.35, $0.1
**Operation of benchtop heater**	$0.04 for 30 mins
**Estimated cost for 9 cm^2**	$1.32
**Estimated cost (cm^{-2})**	~$0.15

Supplementary Table 2. **Comparison of key physicochemical properties between light crude oil and mineral oil used as feed solution.**

Specification	Light crude oil	Mineral oil (Fork w2.5 Motorcycle oil)
Density (kgL^{-1})	0.825	0.827
Viscosity (mm^2s^{-1})	5.96	6.74

Supplementary Table 3. **Composition analysis of sea water from Sydney harbour**

Parameter	Seawater	Parameter	Seawater
pH	8.19 ± 0.00	Sulfate (mg/L SO_4^{2-})	2,964.57 ± 4.42
Conductivity (mS cm^{-1})	56.73 ± 0.01	Chloride / Sulfate ratio	6.91 ± 0.07
Total dissolved salts (TDS, mgL^{-1})	38,576.12 ± 3.83	Total coliforms (cfu/100 ml)	< 1
Turbidity	0.20 ± 0.02	Ecoli faecal bacteria (cfu/100 ml)	< 1
TOC (mgL^{-1})	16.12 ± 1.33	UV_{254} (cm^{-1})	0.014 ± 0.002
$SUVA_{254}$ ($Lmg^{-1}m^{-1}$)	0.0867 ± 0.004	Aluminum (mgL^{-1})	0.011 ± 0.001
Alkalinity (mgL^{-1} $CaCO_3$ equivalent)	120.52 ± 1.16	Arsenic (mgL^{-1})	< 0.001
Water hardness (mgL^{-1} $CaCO_3$ equivalent)	6,461.23 ± 2.49	Cadmium (mgL^{-1})	< 0.001
Phosphate (mgL^{-1} P)	0.007 ± 0.000	Chromium (mgL^{-1})	0.003 ± 0.000
Nitrate (mgL^{-1} N)	0.007 ± 0.001	Nitrite (mgL^{-1} N)	<0.001
Ammonia (mgL^{-1} N)	0.039 ± 0.004	Copper (mgL^{-1})	0.021 ± 0.003
Sodium (mgL^{-1})	12,501.21 ± 12.47	Iron (mgL^{-1})	0.312 ± 0.011
Potassium (mgL^{-1})	405.20 ± 1.35	Manganese (mgL^{-1})	0.005 ± 0.000
Calcium (mgL^{-1})	417.23 ± 2.16	Nickel (mgL^{-1})	< 0.001
Magnesium (mgL^{-1})	1,316.75 ± 3.79	Lead (mgL^{-1})	< 0.001
Sodium Absorption Ratio (SAR)	67.60 ± 2.62	Zinc ($mg^{-1}L$)	< 0.001
Chloride (mgL^{-1})	20,505.33 ± 34.30	Silicon (mgL^{-1})	2.35 ± 0.29

Supplementary Table 4. Details of the intramolecular and intermolecular force potentials used in the Molecular Dynamics simulations.

**Details of intramolecular bonding of SDS molecule [1]**		
Bond length: harmonic potential $E_{bondlength} = K_b(r - r_0)^2$		
**Bond type**	$K_b\left(\frac{Kcal}{mol \cdot Å^2}\right)$	r_0 (Å)
CH_n-CH_n	620	1.53
CH_2-O*	600	1.42
O*-S (O* in CH_2-O*-S)	600	1.58
O-S (O in SO_3)	900	1.46
Bond angle: harmonic potential $E_{angle} = K_a(\theta - \theta_0)^2$		
**Angle type**	$K_a\left(\frac{Kcal}{mol \cdot rad^2}\right)$	$\theta_0(deg)$
CH_n-CH_n-CH_n	124.3	111
CH_2-CH_2-O*	124.3	109.5
CH_2-O*-S	124.3	112.6
O*-S-O	102	102.6
O-S-O	102	115.4

Dihedral angle: Ryckeart and Bellemans potential $E_{dihedral} = \sum_{k=0}^{k=5} c_k \cos^k \varphi$						
	$C_0\left(\frac{kcal}{mol}\right)$	$C_1\left(\frac{kcal}{mol}\right)$	$C_2\left(\frac{kcal}{mol}\right)$	$C_3\left(\frac{kcal}{mol}\right)$	$C_4\left(\frac{kcal}{mol}\right)$	$C_5\left(\frac{kcal}{mol}\right)$
All dihedral angles	2.2176	2.905	-3.136	-0.731	6.271	-7.527

**Details of intermolecular Lennard-Jones and coulombic interactions [1, 2]** $E_{LJ} = 4\epsilon\left[\left(\frac{\sigma}{r}\right)^{12} - \left(\frac{\sigma}{r}\right)^{6}\right]$ **and** $E_c = \frac{Cq_iq_j}{\varepsilon r}$			
**Atoms**	σ (Å)	$\epsilon\left(\frac{kcal}{mol}\right)$	$q(e)$
O (in H_2O)	3.164	0.1628	-1.0484
H	0	0	0.5242
Na+	2.452	0.1684	1
Cl-	5.520	0.01166	-1
C	3.400	0.08600	0
CH_3	3.905	0.175	0
CH_2	3.905	0.118	0
CH_2 (in CH_2-O-S)	3.905	0.118	0.137
S	3.550	0.25	1.2840
O* (O* in CH_2-O*-S)	3	0.17	-0.459
O in (SO_3)	3.15	0.2	-0.654

Supplementary Table 5. **Comparison of surface free energy of permeable graphene and pristine PTFE membrane.**

	γtot [mN/m]	γd [mN/m]	γp [mN/m]	sqrt(γ+)	sqrt(γ-)
Permeable Graphene	36.869	34.585	2.284	2.066	0.631
Pristine PTFE membrane	12.359	9.375	2.984	1.285	1.732

Supplementary References

1. Tummala N R, Striolo A. Role of Counterion Condensation in the Self-Assembly of SDS Surfactants at the Water−Graphite Interface. *The Journal of Physical Chemistry B* **112**, 1987-2000 (2008).

2. Ang E Y M, Ng T Y, Yeo J, Lin R, Geethalakshmi K R. Nanoscale fluid mechanics working principles of transverse flow carbon nanotube membrane for enhanced desalination. *International Journal of Applied Mechanics*, (2017).
