# Supplementary material for: Anti-fouling graphene-based membranes for effective water desalination
Source: Nat Commun. 2018 Feb 14;9:683. doi: 10.1038/s41467-018-02871-3 (PMC5813009; doi:10.1038/s41467-018-02871-3)
Supplement: Supplementary file 2 — Description of Additional Supplementary Files [file 41467_2018_2871_MOESM2_ESM.pdf]

Description of Additional Supplementary Files

File Name: Supplementary Movie 1
Description: Simulation of water molecules permeation through overlapping of graphene grain boundaries.

File Name: Supplementary Movie 2
Description: Molecular Dynamics simulation of single SDS Molecule with Saline water mixtures via permeable graphene with channels.

File Name: Supplementary Movie 3
Description: Molecular Dynamics simulation of bi SDS Molecule with Saline water mixtures via permeable graphene with channels.
